# Comprehensive evaluation of groundwater quality and drought susceptibility in Jazmurian Basin Iran using integrated statistical GIS analysis

**DOI:** 10.1038/s41598-025-95839-5

**Published:** 2025-03-31

**Authors:** Sakineh lotfinasabasl, Azade Gohardoust, Fatemeh Dargahian, Samira Zandifar

**Affiliations:** Agricultural Research, Education and Extension Organization (AREEO), Research Institute of Forest and Rangelands (RIFR), Tehran, Iran

**Keywords:** Arid regions, Spatial salinity dynamics, Drought vulnerability, Cluster and factor analysis, Iran groundwater, Environmental sciences, Environmental monitoring, Hydrogeology

## Abstract

Arid regions face a dual threat of declining groundwater quantity and quality. This study examines these interconnected challenges in the diverse aquifers of Iran’s Jazmurian Basin using GIS and statistics. The study reveals significant spatial variations in salinity, hardness, and sodium content. Of particular concern is the rising electrical conductivity (EC) across most aquifers, especially in the east, which indicates potential water quality degradation. The relationship between groundwater level and salinity is intricate and requires site-specific management. Cluster analysis has identified three distinct groundwater profiles: deep formations that require geological understanding for salinity control, high-quality zones that need recharge protection, and areas that struggle with salinity sources that require identification. Factor analysis identifies salinity and aridity, mineral content related to rock weathering and agriculture, recharge zones requiring protection, and complex interactions between specific ions and groundwater level as key drivers. This study highlights the importance of implementing comprehensive management strategies considering spatial variations, temporal trends, and unique drivers. Targeted interventions, sustainable water use, and effective monitoring programs are essential for safeguarding this vital resource in drought-prone regions. Further research is necessary to refine our understanding of human influences and unique geochemical processes shaping each aquifer system, ultimately enhancing global arid zone groundwater management.

## Introduction

Groundwater, a vital yet finite resource, sustains ecosystems, communities, and life^1–3^. Supporting two-thirds of the global population’s water needs^4^, it faces mounting threats due to rising demand and human activities like land-use changes, urban sprawl, and pollution^5,6^. The sustainable management of groundwater requires understanding its quantity and quality.

Groundwater, stored in soil and rock, is replenished mainly by rainfall^7,8^. Its availability depends on the aquifer size and type, impacting domestic, agricultural, and industrial^6^. Insufficient groundwater leads to water scarcity, whereas excess can cause flooding and waterlogging^9^.

Equally critical groundwater quality is often compromised by human activities. Contaminated water, which harbors hazardous chemicals and pathogens, poses significant health and environmental risks^2^. Excessive extraction, agricultural practices, and industrial pollution are the primary culprits^10,11^. Overpumping lowers water tables, leading to subsidence and saltwater intrusion^12,13^. Agricultural runoff introduces pollutants such as pesticides and fertilizers, jeopardizing water quality for various uses^14^.

Innovative tools, such as Geographic Information Systems (GIS), are crucial for effective management. GIS analysis visualizes contamination patterns, evaluates management strategies, and identifies areas at risk^15–20^. Spatial GIS analysis maps water resources and identifies contamination zones, enabling proactive groundwater protection^21–24^.

This study focuses on the challenges faced by groundwater resources in Iran, a country grappling with aridity and declining water levels. Iran, particularly in its eastern and southern regions, has witnessed alarming declines in groundwater resources due to droughts, increasing water demand, and human activities such as agricultural expansion, land-use change, and urbanization^25^. This study utilizes GIS and other analytical tools to comprehensively evaluate the challenges of sustainable groundwater management in arid environments, specifically in the Jazmurian Basin of southeastern Iran.

The region faces the dual challenge of significant groundwater depletion and degradation. Our study comprehensively addresses these issues by evaluating quantitative and qualitative changes in the basin’s groundwater resources. We examine the physicochemical properties, geochemical composition, and underlying mechanisms that govern groundwater characteristics across its diverse aquifer systems.

We employ a multifaceted investigation, utilizing a robust statistical framework and advanced Geographic Information System (GIS) techniques. we aim to gain valuable insights into the current state of groundwater resources by analyzing extensive data spanning 18 years. We use techniques such as trend analysis, correlation analysis, and statistical analysis to identify areas particularly vulnerable to water scarcity and compromised quality. Additionally, we utilize methods like geospatial clustering and spatial interpolation to understand the basin’s aquifers at both the sub-basin level and the whole.

This study will serve as the foundation for developing effective management strategies for this critical resource in the region^26,27^.

## Study area

**Fig. 1:**
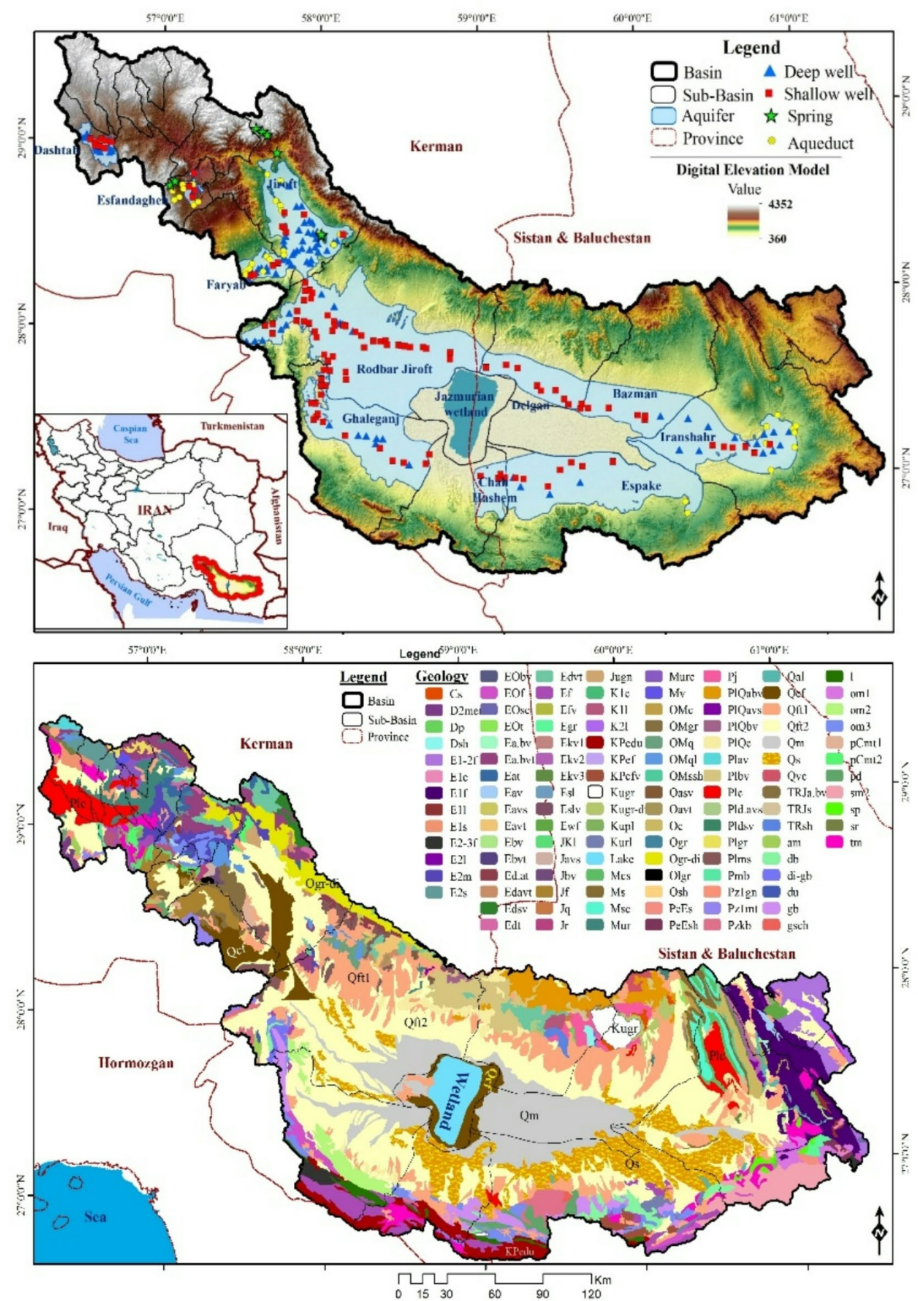
(**a**) Visual Guide to the Jazmurian Basin’s Topography, Aquifers, and Measurement Resources. (**b**) Geological Map of the Jazmurian Basin, Iran. Maps generated using ArcGIS 10.8.1 (https://www.esri.com/en-us/arcgis/products/arcgis-desktop/overview).

The Jazmurian Basin, a vast 69,691 km^2^ arid region in southeastern Iran, boasts diverse landscapes ranging from towering mountains (4,200 m ASL) to arid plains (360 m ASL). The ephemeral Hamun-e-Jazmurian Lake lies at its heart (Fig. 1a).

The basin encounters varying levels of rainfall, with the northern region receiving 400–500 mm per year, whereas the eastern and southwestern parts receive a modest 100–150 mm annually. The drought’s severity is worsened by an imbalance and high evaporation rates ranging from 1,300 to 3,750 mm^28,29^.

Aridisols and Entisols are the dominant soil types. The Halil Rood and Bumper rivers flow through the landscape, while rangelands and salty lands characterize the land cover^30^.

Within the basin, there are a total of 21 sub-basins, some of which lack both plains and aquifers. The alluvial aquifers cover an area of 2,225 km^2^, with the largest single aquifer, covering an area of 5,835 km, located in the Roodbar Jiroft sub-basin. Unconfined aquifers characterize the basin predominantly, with a small confined aquifer region within the Jiroft sub-basin.

The Jazmurian Basin’s contrasting hydrogeology and topography, encompassing mountains, lakes, and diverse formations, presents a unique opportunity to study the interplay between climate, geology, and water resources in an arid environment. This complex tapestry offers valuable insights for understanding and managing these regions.

## Geological setting

The diverse array of formations characterizes the geology of the Jazmurian Basin, including shale, limestone, dolomite, conglomerate, marl, and gypsum. Shale, while offering limited water potential, is a significant component of the basin’s stratigraphy. Limestone and dolomite formations contribute to the region’s aquifer systems, providing essential groundwater storage and flow pathways. Conglomerate deposits, often found in alluvial fans, enhance the basin’s hydrogeological complexity.

Marl and gypsum formations are particularly noteworthy due to their porosity, which makes them promising for groundwater storage. However, these formations are also susceptible to erosion, impacting water quality and availability. High- and low-level Piedmont fan deposits dominate the basin, which is crucial for groundwater recharge. Valley terrace deposits further contribute to the basin’s hydrogeological framework, providing additional storage and flow pathways.

Interspersed within these geological formations are wetlands and sand sheets, which play a vital role in the basin’s hydrology. The unique climate of the region, characterized by arid and semi-arid conditions, leads to the formation of salt and clay deposits. These deposits significantly influence the hydrogeology of the basin, affecting both groundwater quality and availability (Fig. 1b).

Overall, the geological diversity of the Jazmurian Basin, combined with its unique climatic conditions, creates a complex and dynamic hydrogeological system that requires careful management and study.

## Material and method

This study employed a multifaceted approach to unravel the key drivers impacting groundwater quality within the Jazmurian Basin. Data analysis encompassed various statistical and chemical techniques, offering a comprehensive understanding of the complex interplay of factors influencing water chemistry.

### Data acquisition and processing

This study focuses on 11 specific sub-basins within the region: Dashtab, Esfandaghe, Faryab, Jiroft, Roodbar Jiroft, Ghaleganj, Chah Hashem, Espakeh, Bazman, Delgan, and Iranshahr. These 11 sub-basins are the only ones in the region that harbor aquifers.

#### Data source

A comprehensive dataset covering 18 years, from 2000 to 2018, has been obtained from the Ministry of Energy. The dataset comprised quantitative and qualitative data from 256 groundwater resources located within 11 sub-basins of the region (Fig. 1). These resources included both confined and unconfined aquifers, consisting of deep wells (96), shallow wells (115), aqueducts (37), and springs (8). Water quality data collection commenced in 2000, and 4,324 water samples were collected throughout the study period (Fig. 1).

### Data used

The collected water samples were analyzed for their groundwater quality through assessments, as per APHA^31^ guidelines. Analyzed physicochemical parameters included electrical conductivity (EC), total dissolved solids (TDS), pH, major cations (calcium, magnesium, sodium, and potassium), significant anions (chlorine, sulfate, and carbonate), sodium percentage (Na %), sodium absorption ratio (SAR), and total water hardness (TH). The data on water level was utilized to evaluate the groundwater fluctuation.

### Data quality control

We have implemented strict quality control measures to ensure the results are error-free.

1. Ionic Balance Check: Data points with an ionic balance error exceeding 5% were excluded (Eq. 1)^32^.
2. Consistency Evaluation: To compare EC, TDS, and TC, established relationships (Eq. 2 and Eq. 3) were used^32^.
3. Parameter Calculations: Standard formulas (Eqs. 4, 5, and, 6) were used to calculate SAR, Na%, and TH^32^.

1 $$\:Error\%=\frac{\sum\:TC\:\left(total\:cations\right)-\sum\:TA\:\left(total\:anions\right)}{\sum\:TC+\sum\:TA}*100$$

2 $$\:\frac{TDS}{EC}=0.55-0.76\:$$

3 $$\:\frac{EC}{TC}=\:90-110$$

4 $$\:SAR=\frac{Na}{\sqrt[2]{Ca+Mg}}$$

5 $$\:Na\%=\left(\right(Na+K)/Na+K+Ca+Mg\left)\right)*100$$

6 $$\:TH\left(mg/l\right)={Ca}_{(mg/l)}*2.497+{Mg}_{\left(mg/l\right)}*4.118$$

### Data analysis

#### Statistical analysis

- Normality testing: The normality distribution of the data was done using QQ and box plots in IBM SPSS Statistics 27.
- Temporal and Spatial Trends: Mann-Kendall trend tests examined changes in average EC over time within each aquifer. Pearson correlation analysis explored relationships between physicochemical parameters.

#### Spatial analysis

Kriging maps visualized the spatial distribution of groundwater parameters using a geostatistical approach in ArcGIS 10.8.1 software^33,34^.

The Study of groundwater drought: An investigation was carried out using the Groundwater Risk Index (GRI) developed by Mendicino et al.^35^ and refined by subsequent studies^36–39^ to evaluate potential vulnerability to environmental changes and stressors concerning groundwater drought. The GRI measures the difference between current groundwater levels and long-term average levels. This helps determine if there is a potential drought and the associated risk to water quality. The GRI is calculated using Eq. (7), which considers the groundwater level at a given time (Zt) and the long-term mean groundwater level (Zm). Negative GRI values signify drought conditions, and a higher negative represents the risk of water quality degradation. Conversely, positive GRI values represent wetter conditions and reduced vulnerability to water quality issues. Thus, the GRI is a valuable tool for understanding the relationship between groundwater availability and potential risks to water quality in a given region. Table 1 provides a guide on how to interpret GRI values.

7 $$\:GRI=((Zt-Zm)/Zm)$$

**Table 1:** Classification of groundwater drought according to the GIR index.

GRI	Classification	Condition
GRI ⪰ 2.00	Extremely Wet	Indicating exceptionally high groundwater levels, potentially causing flooding or waterlogging issues.
1.50 ≤ GRI ≤ 1.99	Very Wet	Indicates significantly elevated groundwater levels
1.00 ≤ GRI ≤ 1.49	Moderately Wet	Suggests groundwater levels above average, but not excessively high
0.5 ≤ GRI ≤ 0.99	Mild wet	
− 0.49 ≤ GRI ≤ 0.49	Normal	Represents typical groundwater conditions, with levels within a balanced range
-0.50 ≤ GRI ≤ -0.99	Mild Dry	Points to below-average groundwater levels, signaling the onset of drought conditions.
-0.1 ≤ GRI≤ -1.49	Moderately Dry	
-1.5 ≤ GRI ≤ -1.99	Severely Dry	Marks a significant depletion in groundwater, leading to severe drought and associated impacts.
GRI ≤ -2.00	Extremely Dry	Denotes critically low groundwater levels, often resulting in widespread and acute drought conditions.

### Exploring key factors

To comprehensively understand the key factors influencing groundwater quality, a multi-pronged approach was adopted:

- *Gibbs diagrams*: This geochemical method visually represented the hydro-chemical facies and dominant water-rock interaction processes controlling groundwater composition. By plotting major ions on a specific plot, the influence of evaporation, precipitation, and mineral dissolution was elucidated^40^.
- *Cluster analysis*: This statistical method grouped groundwater samples based on physicochemical properties, unveiling spatial variations in water quality and influencing factors. Widely used in environmental studies, cluster analysis identifies similarities and differences among water quality parameters, fostering a better understanding of the underlying processes affecting groundwater systems^41–43^.
- *Factor analysis (FA)*: This statistical technique statistically identified underlying patterns and correlations within the physicochemical data. By reducing data into smaller, meaningful factors, FA revealed the significant processes governing groundwater quality across the diverse geochemical landscapes of the 11 aquifers^44–48^.

### Integration and interpretation

Findings from the various analyses were integrated to provide a holistic understanding of the key drivers impacting groundwater quality within the Jazmurian Basin. The combined insights from Gibbs diagrams, factor analysis, cluster analysis, and statistical analyses offered a comprehensive picture of the complex interplay between natural and anthropogenic factors shaping water chemistry across the diverse aquifer systems.

## Results and discussion

### Assessment of the physicochemical parameters of groundwater

Assessing physical and chemical properties in groundwater is essential for determining its quality (Table 2). These properties provide valuable information about the water’s characteristics and help determine its suitability for various purposes, such as drinking, irrigation, and industrial use. Electrical Conductivity (EC) and Total Dissolved Solids (TDS) indicate water bodies’ mineral content and salinity. pH indicates the acidity or alkalinity of water, and an imbalance in pH levels may suggest the presence of contaminants or natural processes that can affect water quality. SAR measures the potential for soil degradation, while Neil is essential for assessing soil salinity, water suitability for irrigation, and the risks associated with sodium-induced dispersion in soils. TH determines water hardness, and is necessary for optimal water use, preventing scaling, and maintaining equipment efficiency. The concentrations of cations (such as sodium, potassium, calcium, and magnesium) and anions (such as chloride, sulfate, nitrate, and fluoride) are crucial for identifying specific contaminants and potential risks to human health. Elevated levels of certain cations or anions may indicate pollution or natural processes affecting groundwater safety. The statistical summary of the physicochemical parameters of the aquifers is presented in Table 2.

#### Electrical conductivity (EC)

It is crucial to analyze the electrical conductivity (EC) values to understand the impact of salinity on water quality and the surrounding ecosystem. These values serve as an indicator of the level of salinity in water bodies. Among different sub-basins, Bazman has the highest mean EC value of 4597 µS/cm, indicating relatively high salinity in the water. On the other hand, Esfandaghe has the lowest mean EC value of 809 µS/cm, suggesting less saline water than other sub-basins. Roodbar Jiroft has the maximum EC value of 11,100 µS/cm, indicating significant salinity in the water. Conversely, Jiroft has the lowest EC value of 170 µS/cm, indicating that the water in this sub-basin has the least salinity.

#### Total dissolved solid (TDS)

The sub-basin with the highest average TDS value is Bazman, which has an average TDS value of 2962 mg/L indicating a relatively high level of dissolved solids in the water. On the other hand, the sub-basin with the lowest average TDS value is Esfandaghe, which has an average TDS value of 528 mg/L. The water in Esfandaghe has the lowest dissolved solids content compared to other sub-basins. The maximum TDS value was found in Roodbar Jiroft, reaching 7215 mg/L, which indicates a high level of dissolved solids in the water. Meanwhile, Jiroft had the lowest TDS value of 113 mg/L, indicating the lowest dissolved solids content.

#### pH (acidity)

The sub-basin with the highest average pH value is Espakeh, with an average pH value of around 7.9, indicating that the water is slightly alkaline. The aquifer with the lowest average pH was Jiroft, with an average pH of about 6.4. Jiroft water is moderately acidic. The maximum pH values of Espakeh and Roodbar Jiroft were 9.4 and 9.3, respectively. This high pH indicates alkalinity. The sub-basins with the lowest pH values are Ghaleganj and Bazman, with minimum pH values around 6.2 and 6.3, respectively.

#### Sodium adsorption ratio (SAR)

Espakeh has the highest average SAR value of 16.6, indicating a significant risk of sodium in the water for irrigation. In contrast, Esfandaghe has the lowest average SAR value of about 2.6, indicating a low sodium risk. It is worth noting that the SAR value in Espakeh reaches around 47.6, the highest among all sub-basins. The SAR value of the water indicates the risk of soil dispersion and reduced permeability if used for irrigation. Jiroft has the lowest SAR value, approximately 0.1, and therefore poses a minimal sodium risk.

#### Sodium percentage (Na%)

The sub-basin with the highest average Na% value is Espakeh, with a percentage exceeding 89%, which means a significant amount of sodium ions in the water. On the other hand, the sub-basin with the lowest average Na% value is Esfandaghe, with a percentage of approximately 44.2%, indicating that the water in this sub-basin has the lowest sodium content. It is important to note that the maximum Na% value is found in Espakeh, reaching 98%. This value indicates a significant risk of soil dispersion and reduced permeability for irrigation purposes. In contrast, Jiroft’s water poses minimal sodium hazard and has the lowest Na% value, with a minimum of approximately 0.1% across all the sub-basins.

#### Total hardness (TH)

Bazman, among the sub-basins, has the highest average TH value, approximately 368.8 meq/L, indicating a relatively high hardness. It is important to note that all statements are presented objectively and without bias. On the other hand, Espakeh has the lowest mean TH value, approximately 64 meq/L, indicating the minimum hardness. The maximum TH value is found in Ghaleganj, reaching 3930 meq/L, which indicates significant mineral content in the water. The sub-basin with the lowest TH value is Espakeh, with a minimum TH of approximately 14.9 meq/L, indicating the water has minimal hardness.

#### Cations and anions

The Bazman had the highest average sodium, calcium, and potassium cations. It also had the highest average values of chloride and sulfate anions.

The Roodbar Jiroft had the highest average value of magnesium. The Esfandaghe and Espakeh had the lowest average values of sodium and calcium, respectively. The Espakeh had the highest average values of carbonate and bicarbonate. Consequently, the Bazman had high cation concentrations, while the Roodbar Jiroft had high magnesium levels. The Esfandaghe and Espakeh had lower cation values, and the Espakeh had high carbonate and bicarbonate levels. These findings provide valuable information about the chemical composition of these sub-basins.

**Table 2:** Statistical summary of the physicochemical parameters of the aquiferss in the study area.

Sub-basin	value	EC (µS/cm)	TDS (mg/L)	pH	SAR	Na (%)	Th (mg/L)	Na^+^	Ca^2+^	Mg^+ 2^	K^+^	HCO_3_^−^	CO_3_^2−^	Cl^−^	SO_4_^2−^
								meq/L							
Espakeh	Mean	1495	972	7.9	16.6	89.1	63.8	11.75	0.7	0.58	0.13	3.93	0.14	5.78	3.41
	Max^*^	3950	2568	9.4	47.6	98	208.3	32.4	1.9	2.82	0.96	9.2	3	14.50	15.7
	Min^**^	513	333	6.4	4.4	60.7	14.9	3.7	0.19	0.1	0.05	0.5	0	0.5	0
	SD^***^	588.65	382.62	0.44	8.40	7.23	37.93	5.24	0.44	0.51	0.08	1.81	0.42	2.46	3.30
Esfandaghe	Mean	809	528	7.7	2.6	44.2	220.7	4.09	2.77	1.67	0	3.62	0	1.83	3.05
	Max	2250	1463	8.4	8.2	76.5	597.2	14.5	8.00	5.50	0	6.5	0	10.8	13.1
	Min	360	234	6.8	0.5	14.3	114.6	0.8	1.20	0.4	0	2	0	0.3	0.2
	SD	471.11	306.66	0.34	1.48	11.54	99.79	3.19	1.26	1.03	0.00	0.73		1.87	3.06
Iranshahr	Mean	2362	1531	7.6	11.6	79.6	201.7	17.23	2.15	1.91	0.23	3.74	0.02	11.47	6.36
	Max	8300	5395	8.7	28.1	94.2	809.6	64.50	7.80	8.5	0.90	12.2	2.50	54.9	32.4
	Min	509	331	6.3	2.3	47.4	44.9	2.75	0.20	0.07	0.05	0.4	0	1.20	0
	SD	1691.44	1091.87	0.35	5.34	6.64	139.92	12.90	1.38	1.56	0.17	1.64	0.13	10.89	5.08
Bazman	Mean	4597	2962	7.4	18.2	82.6	368.8	34.48	4.81	2.6	0.44	2.64	0	27.99	11.78
	Max	6790	4414	8.1	39.2	95.4	641.9	51.1	10.2	5.7	0.70	4.6	0	47.2	26.5
	Min	2333	541	6.3	7.8	66.1	89.3	15.95	0.7	0.8	0.10	0.5	0	13.8	3
	SD	1263.99	843.53	0.28	3.79	3.47	130.76	9.59	2.31	0.97	0.13	0.72	0.00	9.53	4.06
Jiroft	Mean	859	559	7.6	2.9	45	231	4.28	3.08	1.56	0	3.13	0	2.64	3.14
	Max	3150	2048	9.0	12.7	86.2	846.4	24.60	12.00	7.00	0	6	0	22	13.6
	Min	170	113	6.4	0.1	6.3	39.8	0.10	0.50	0.20	0	1.4	0	0.2	0.00
	SD	459.53	299.08	0.37	2.05	15.70	117.00	3.37	1.62	0.91	0.00	0.78		2.79	2.64
Chah Hashem	Mean	1714	1121	7.7	11.8	81.5	123.7	12.79	1.38	1.11	0.14	2.97	0.04	6.93	5.54
	Max	3550	2308	8.5	23.3	95.5	302.7	25.90	2.80	3.68	0.6	5.9	0.7	12.5	18.7
	Min	380	247	6.9	0.8	26.3	29.8	0.96	0.20	0.30	0	0.2	0	0.8	0.15
	SD	817.58	531.05	0.35	5.59	10.24	54.31	6.42	0.69	0.67	0.11	1.19	0.13	3.48	4.60
Dashtab	Mean	1507	982	7.7	4.2	46.9	364.8	8.33	3.77	3.57	0	3.28	0	5.7	6.65
	Max	4820	3133	8.7	9.3	77	1241.7	30.8	13	13	0	5.4	0	27	34
	Min	350	228	6.7	0.3	10.3	149.2	0.40	1.50	0.8	0	1.5	0	0.4	0.2
	SD	890.05	579.49	0.35	2.64	17.40	201.93	6.44	2.00	2.27	0.00	0.58		5.05	5.23
Delgan	Mean	2829	1839	7.5	14	82.5	222.5	20.72	2.57	1.90	0.26	3.13	0.01	15.3	7.12
	Max	5140	3341	8.3	22.6	92.3	602.9	37.26	9.50	4.37	0.5	6.6	0.4	40.6	18.3
	Min	1056	686	6.8	5.6	71.3	59.6	6.40	0.50	0.60	0.1	0.4	0	1.6	0
	SD	836.60	543.79	0.28	2.94	3.83	93.66	6.04	1.59	0.79	0.09	1.47	0.06	6.11	3.53
Roodbar Jiroft	Mean	2173	1417	7.6	9	70.3	345.3	16.06	4.32	2.62	0	3.46	0	10.48	9.03
	Max	11,100	7215	9.3	26.8	92.6	2587.4	96	34	180	0.2	9.5	0	100	49.7
	Min	549	358	6.4	2	32.8	24.9	3.1	0.4	0.1	0	1	0	1.2	0
	SD	1219.29	782.44	0.41	3.83	10.30	254.65	9.77	3.44	2.23	0.02	1.04	0.00	8.76	5.72
Faryab	Mean	1020	664	7.7	5.1	60.3	194.4	6.76	2.45	1.46	0	3.57	0	3.25	3.83
	Max	3750	2438	8.9	13.8	88.4	606.5	29.20	9.00	5.50	0	7.00	0	23	15
	Min	450	293	6.6	1.3	27.3	39.8	1.60	0.40	0.20	0	1.20	0	0.5	0.6
	SD	517.08	336.63	0.37	2.96	16.06	98.03	4.50	1.29	0.91	0.00	1.02		2.91	2.45
Ghaleganj	Mean	2069	1351	7.7	11.2	76.3	285.6	16.14	3.83	1.91	0	3.51	0	10.05	8.32
	Max	1015	6598	8.7	30	93.8	3930.1	65	56	30	0	8.1	0	88	50
	Min	611	397	6.2	2.2	32.3	49.7	3.2	0.50	0.2	0	0.8	0	1.8	0.7
	SD	1490.25	978.20	0.48	5.81	12.70	482.05	10.52	6.71	3.14	0.00	1.06		11.04	6.82
Jazmurian Basin	Mean	1699	1102	7.65	7.94	64.39	251.79	11.86	3.12	1.94	0.06	3.42	0.04	7.61	5.9
	Max	11,100	7215	9.4	47.6	98.0	3930.1	96.00	56.00	30.0	0.96	12.20	3.00	100.00	50.0
	Min	170	113	6.2	0.12	6.3	14.9	0.10	0.19	0.07	0.00	0.20	0.00	0.20	0.0
	SD	1326.95	859.17	0.39	6.04	19.54	226.60	10.51	3.04	1.83	0.13	1.14	0.20	8.87	5.19

*Max: Maximum, **Min: Minimum, ***SD: standard deviation.

### Uncovering salinity dynamics in the Jazmurian basin: spatial variations, temporal trends, and fluctuations of electrical conductivity

**Fig. 2:**
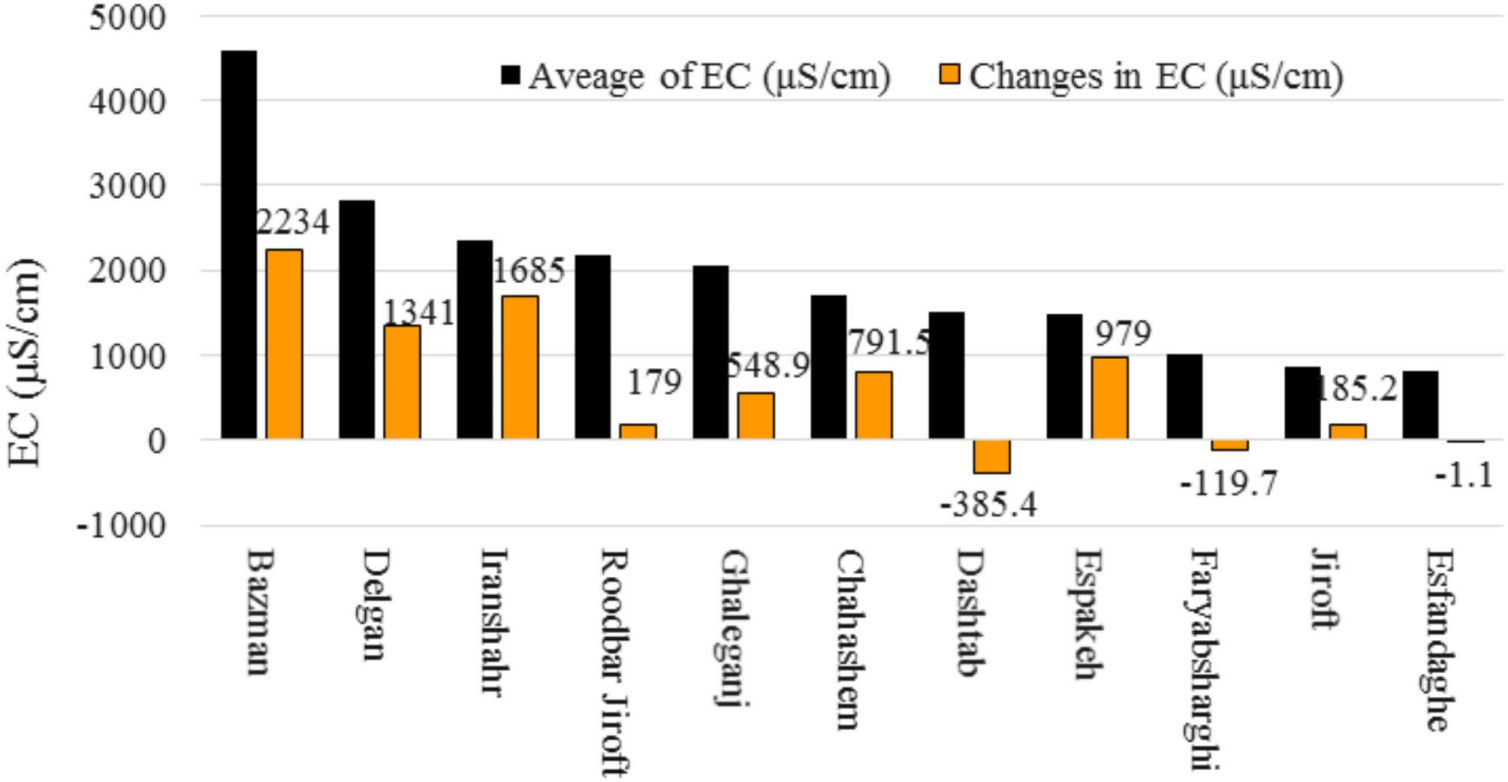
The average electrical conductivity (EC) and its changes across different aquifers.

The data analysis showed that the average EC concentration in the aquifers of the Jazmurian Basin ranged from 809.2 to 4597 µS/cm, with an average of 1699 µS/cm (Fig. 2). This wide range indicates significant variability in salinity and mineral content across the different aquifers. The highest average EC value was observed in Bazman, located in the eastern part of the basin, indicating a higher concentration of dissolved solids in its groundwater. In contrast, Esfandaghe, situated in the western part of the basin, has the lowest average EC value of all the aquifers studied exploring groundwater and has relatively lower levels of dissolved solids than the other areas within the Jazmurian Basin.

Furthermore, an analysis of changes in groundwater EC concentrations in different aquifers revealed distinct patterns. Specifically, the Dashtab, Faryab, and Esfandaghe aquifers exhibited a significant decrease in concentration. In contrast, the other aquifers displayed a rise in concentration, as shown in Fig. 2. To establish the direction and significance of these changes, we conducted the Mann-Kendall trend test, and Table 3 represents the results.

Table 3 displays the outcomes of the Mann-Kendall trend test, revealing noteworthy upward trends in salinity levels across various regional aquifers. This pattern implies a possible deterioration in water quality. Notably, the Jiroft, Roodbar Jiroft, Ghaleganj, Cha Hashem, Espakeh, Bazman, and Delgan aquifers all exhibit statistically significant increases in salinity. The Dashtab aquifer shows a significant decreasing trend, indicating a reduction in salinity and an improvement in water quality.

The Esfandaghe, Faryab, and Iranshahr aquifers do not display any significant trends. It is worth noting that the trend test did not yield meaningful results for the Iranshahr aquifer despite an overall observed increasing trend. The discrepancy may be due to the consecutive years of 2013 and 2014, which show a decreasing trend. These two years of declining data may have affected the overall trend analysis, leading to the failure to detect a significant trend.

It is crucial to consider such fluctuations or anomalies in the data when interpreting the results of trend tests. Acknowledging these variations can provide a more comprehensive understanding of water quality dynamics in the studied aquifers. The results are consistent with the national trends^49^, indicating a concerning decline in water quality from west to east within the Jazmurian Basin. Specifically, increasing EC and higher salinity concentrations were observed in eastern aquifers. It is important to note that these observations are objective and based on scientific data.

**Table 3:** Mann-Kendall trend analysis of electrical conductivity (EC) in Jazmurian aquifers (2001–2018).

Aquifer	*P*-value (two-tailed)	Sen’s slope	Confidence interval	Trend	Trend type
Dashtab	0.039	− 13.71	− 12.7, − 14.4	Yes	Decreasing
Esfandaghe	0.052	7.04	5.4,7.96	No	Not detected
Faryab	0.490	− 2.69	− 1.9, − 4.3	No	Not detected
Jiroft	0.000	8.46	7.9, 8.8	Yes	Increasing
Roodbar Jiroft	0.002	29.95	25.3, 30.36	Yes	Increasing
Ghaleganj	0.006	40.06	38.06, 43.96	Yes	Increasing
Chah Hashem	0.000	68.99	65.8, 71.67	Yes	Increasing
Espakeh	0.047	30.05	26.77, 30.7	Yes	Increasing
Iranshahr	0.129	39.53	30.7, 46.04	No	Not detected
Bazman	0.002	118.82	112.3, 132.2	Yes	Increasing
Delgan	0.000	139.29	134.05, 147.09	Yes	Increasing

**Fig. 3:**
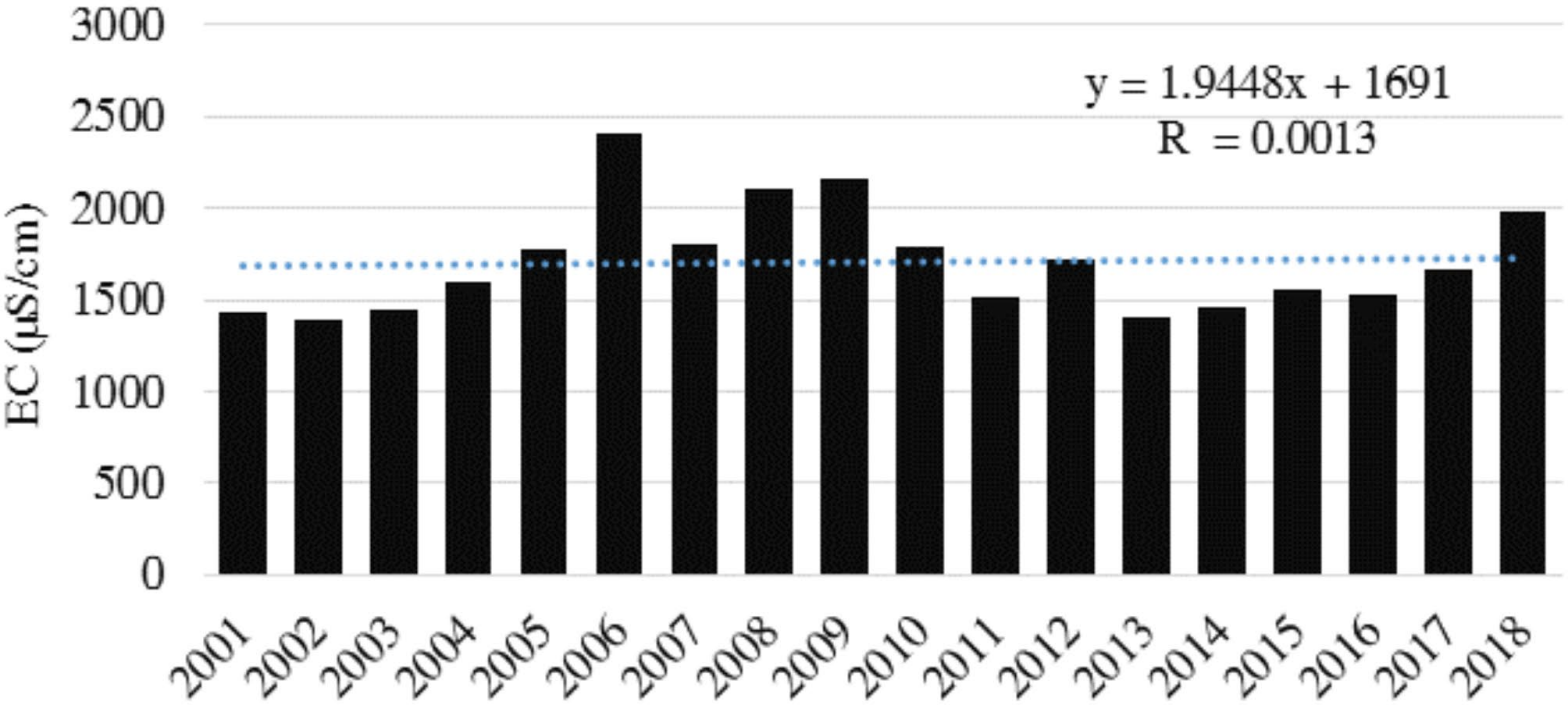
Annual average of electrical conductivity (EC) in Jazmurain Basin (2001–2018).

During the 18-year, the annual average electrical conductivity (EC) of the Basin (Fig. 3) fluctuated significantly, indicating a dynamic system influenced by various factors. The average EC values ranged from a minimum of 1386.57 in 2002 to a peak of 2406 µS/cm in 2006, representing a difference of over 1000 µS/cm. Although some periods demonstrate relative stability, such as 2015–2016, others show sharp increases and decreases, indicating potential salinization or freshening of water sources. It is necessary to investigate the underlying causes of these fluctuations to understand and manage water quality in the region. Figure 3 illustrates the overall fluctuating trend of EC with significant increases and decreases year-on-year. However, there was a net increase throughout the entire period, with the value in 2018 being nearly 550 units higher than in 2001.

**Fig. 4:**
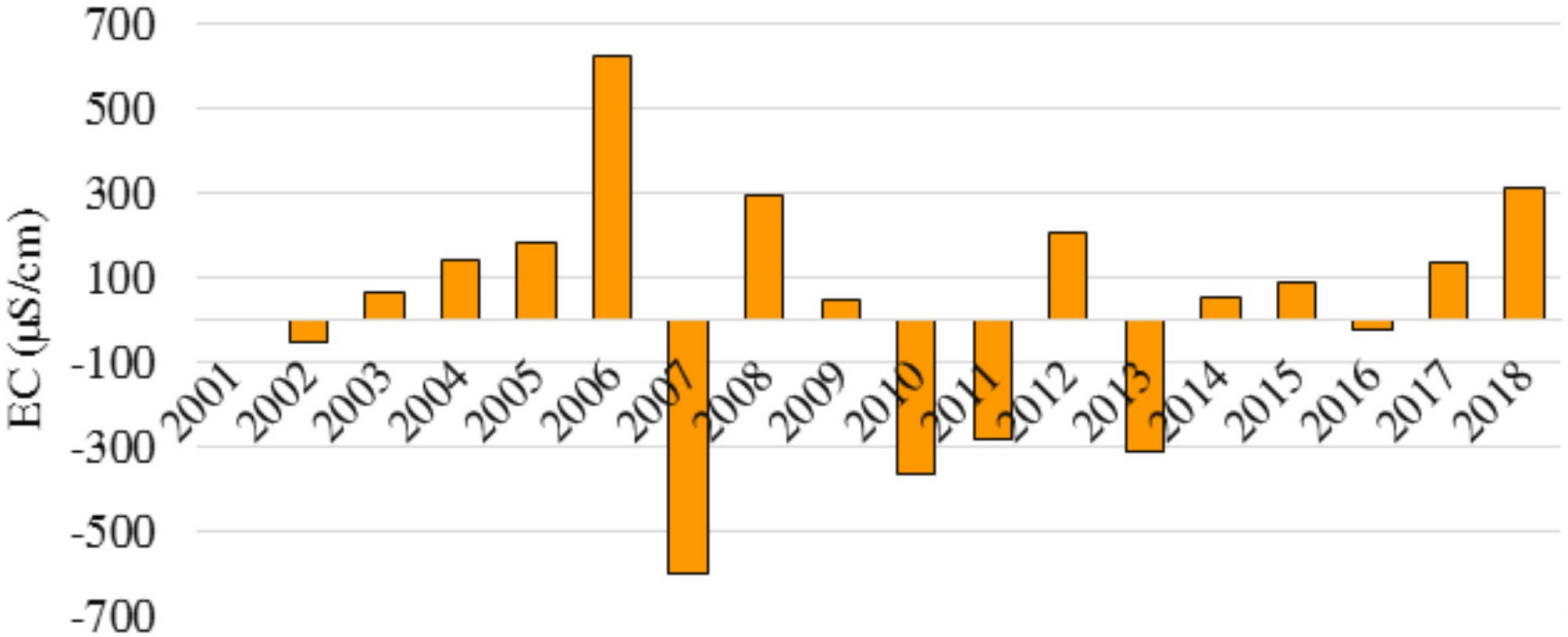
Annual changes in electrical conductivity (EC) in the Jazmurain Basin (2001–2018).

**Fig. 5:**
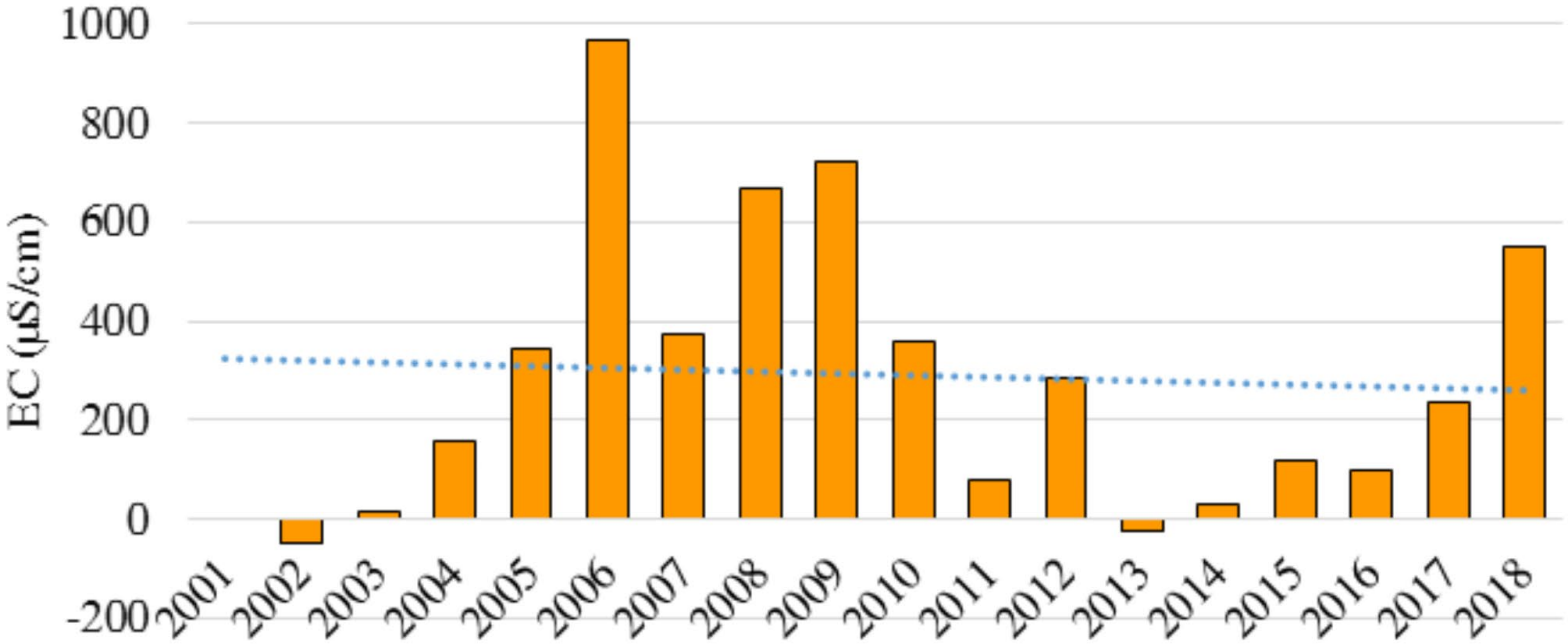
Cumulative sum of annual changes in electrical conductivity (EC) in Jazmurain Basin (2001–2018).

The electrical conductivity (EC) levels showed significant fluctuations over the 18-year, as demonstrated in Fig. 4. In 2006, there was a surge of 627.8 µS/cm, indicating a period of intense salinization. However, in 2007, there was a drop of 598.62 µS/cm, potentially indicating a rapid shift towards fresher water conditions. These significant fluctuations demonstrate the dynamic nature of electrical conductivity (EC) in the region and emphasize the importance of continuous monitoring to comprehend the underlying causes and track future trends. Although the overall change over the 18 years resulted in a net increase of 549.5 µS/cm, the year-to-year variations reveal a complex interplay between salinization and freshening influences. The analysis indicates that the most minor decrease in EC was observed in 2002, with a difference of -49.3 µS/cm, while the smallest increase was 51 µS/cm and occurred in 2009. These nuances emphasize the significance of detailed analysis beyond yearly changes to understand the dynamic behavior of EC in this system. Figure 5 illustrates the Cumulative Sum of Annual Changes in EC, which tracks the overall trend of EC over time. From a negative score in 2002, the EC steadily increased over the next 18 years, reaching a positive 549.5 in 2018. This upward trend underscores the importance of ongoing monitoring to establish long-term patterns and guide effective management strategies.

### Unveiling the ionic drivers of electrical conductivity: Spatial variations and aquifer-specific insights

Monitoring groundwater electrical conductivity (EC) and its correlation with ion levels is crucial for evaluating water quality. Spatial variations in EC can help identify sources of contamination, evaluate the effectiveness of water treatment processes, monitor changes in water suitability for various applications over time, and enable better water management strategies.

To analyze how different ions affect electrical conductivity (EC) values, we examined the temporal correlation between EC and ion concentrations from 2001 to 2018. We employed the Pearson correlation coefficient for this analysis, with a significance level of 5%. The results of this investigation, including the p-values indicating the statistical significance of the observed correlations, are presented in Table 4. Lower p-values, usually below 0.05, indicate more robust evidence for a valid correlation.

Table 4 presents the results of the correlation analysis, which demonstrate that cations and anions, specifically Na^+^, Ca^2+^, Mg^2+^, Cl^−^, and SO_4_^2−^, have the most significant impact on EC concentration. The correlation coefficients between EC and Na^+^, Cl^−^, and SO_4_^2−^ indicate a strong positive linear correlation, while Ca^2+^ shows a moderate positive correlation. Mg and CO_3_^2−^ exhibit a very weak positive correlation, and HCO_3_^−^ shows a weak negative correlation.

**Table 4:** Correlation between electrical conductivity (EC) and ions within the groundwater of the basins.

Variables	Value*	EC	Na^+^	Mg^2+^	Ca^2+^	So_4_^2−^	Cl^−^	HCO_3_^−^	CO_3_^2−^
EC	r	1							
	p-value	0							
Na^+^	r	**0.984**	1						
	p-value	**< 0/0001**	0						
Mg^2+^	r	**0.213**	0.080	1					
	p-value	**0.001**	0.216	0					
Ca^2+^	r	**0.438**	**0.343**	**0.523**	1				
	p-value	**< 0/0001**	**< 0/0001**	**< 0/0001**	0				
So_4_^2−^	r	**0.892**	**0.889**	**0.311**	**0.560**	1			
	p-value	**< 0/0001**	**< 0/0001**	**< 0/0001**	**< 0/0001**	0			
Cl^−^	r	**0.985**	**0.977**	0.142	**0.433**	**0.847**	1		
	p-value	< 0/0001	< 0/0001	0.028	**< 0/0001**	**< 0/0001**	0		
HCO_3_^−^	r	− 0.113	− 0.177	**0.495**	− 0.021	− 0.167	− 0.215	1	
	p-value	0.082	0.006	**< 0/0001**	0.742	0.010	0.001	0	
CO_3_^2−^	r	0.051	0.090	− 0.215	− **0.33**	− 0.040	0.028	0.097	1
	p-value	0.430	0.167	0.001	**< 0/0001**	0.535	0.666	0.134	0

*The given numbers in bold indicate the significant correlation.

Delving into the specifics of spatial correlation, we investigated the interplay between electrical conductivity (EC) and various ions in particular aquifer systems through Table 5 analysis.

**Fig. 6:**
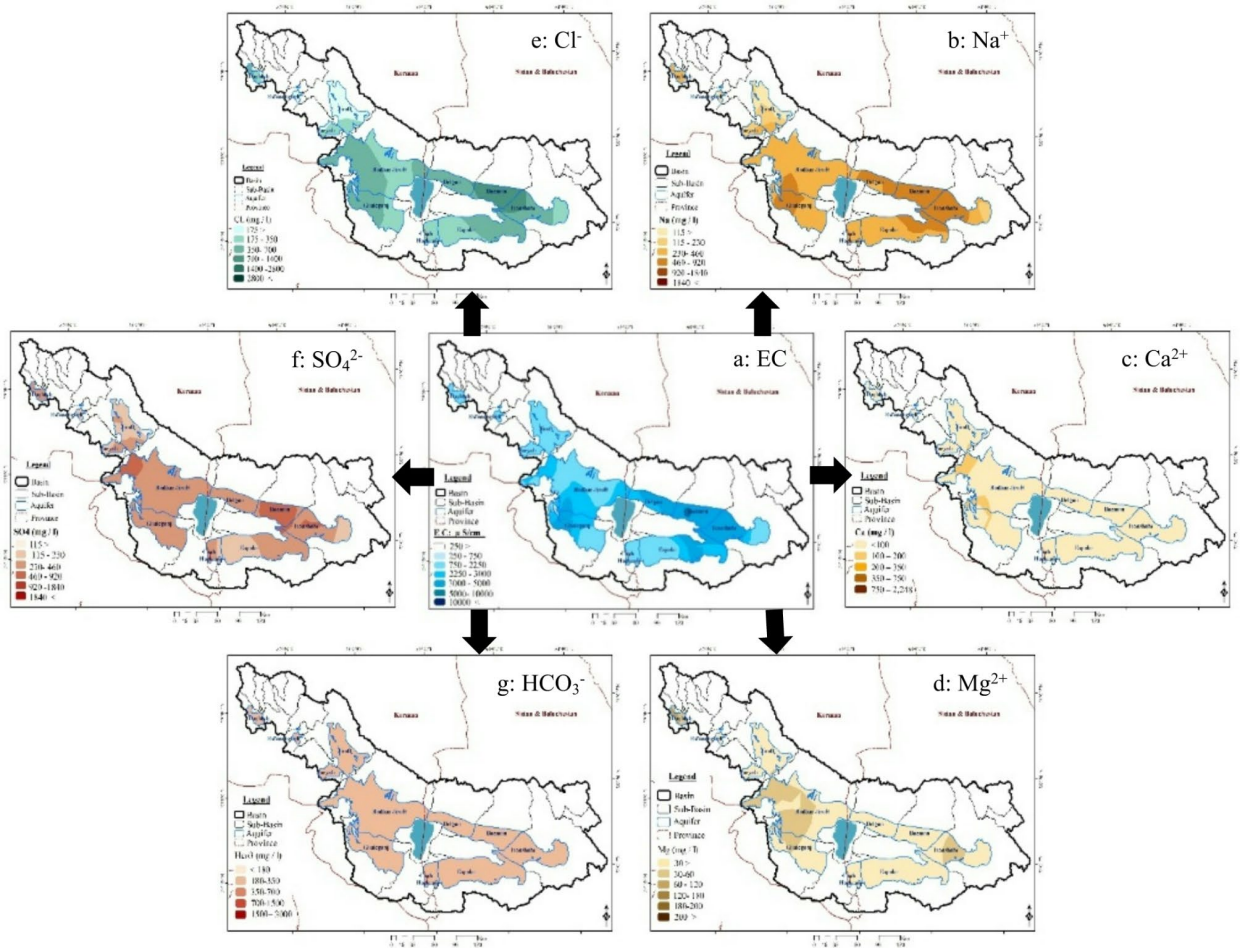
Variation maps of EC (**a**), Na^+^ (**b**), Ca^+ 2^ (**c**), Mg^+ 2^ (**d**), Cl^−^ (**e**), SO_4_^2−^ (**f**) and HCO_3_ ^− 1^ (**g**). Maps generated using ArcGIS 10.8.1 (https://www.esri.com/en-us/arcgis/products/arcgis-desktop/overview).

Additionally, we performed a geospatial investigation using kriging interpolation to generate raster maps for EC and seven essential ions: sodium, calcium, magnesium, potassium, chloride, sulfate, bicarbonate, and carbonate (Fig. 6). To guarantee consistent pixel sizes, prepared maps underwent resampling. The maps were analyzed using a fishnet grid to obtain a tabular dataset linking each zone with average values for EC and each ion.

Pearson’s correlation coefficients were calculated for each ion-EC pair (α = 0.05) in Minitab19 to identify statistically significant relationships and their contribution to observed spatial patterns in EC. Table 5 presents the results of relationships.

This geospatial approach aimed to identify the main ionic drivers of EC variability in the basin and its aquifers, providing insights into the geochemical processes that shape water quality distribution.

**Table 5:** Correlation between electrical conductivity (EC) and ions in the groundwater by aquifers.

Aquifer	Value*	Na^+^	Mg^2+^	Ca^2+^	Cl^−^	SO_4_^2−^	HCO_3_^−^	CO_3_ ^− 2^
Dashtab	R	**0.964**	**0.771**	**0.728**	**0.922**	**0.969**	0.293	
	P-value	**< 0/0001**	**0.00**	**0.001**	**< 0/0001**	**< 0/0001**	0.254	
Esfandaghe	R	**0.806**	0.235	**0.643**	**0.852**	**0.768**	− 0.100	
	P-value	**0.00**	0.382	**0.007**	**< 0/0001**	**0.001**	0.712	
Faryab	R	**0.820**	**0.578**	− 0.088	**0.879**	**0.678**	0.361	
	P-value	**0.00**	**0.019**	0.745	**< 0/0001**	**0.004**	0.170	
Jiroft	R	**0.806**	0.038	**0.777**	**0.920**	**0.766**	− 0.019	
	P-value	**0.806**	0.038	**0.777**	**0.920**	**0.766**	− 0.019	
Roodbar Jiroft	R	**0.922**	0.373	**0.858**	**0.957**	**0.840**	− **0.590**	
	P-value	**< 0/0001**	0.141	**< 0/0001**	**< 0/0001**	**< 0/0001**	**0.013**	
Ghaleganj	R	**0.911**	**0.730**	**0.769**	**0.979**	**0.934**	0.046	
	P-value	**< 0/0001**	**0.001**	**0.000**	**< 0/0001**	**< 0/0001**	0.866	
Chah Hashem	R	**0.975**	0.207	0.246	**0.874**	**0.800**	0.268	0.306
	P-value	**< 0/0001**	0.479	0.397	**< 0/0001**	**0.001**	0.354	0.288
Espakeh	R	**0.973**	− 0.343	− **0.652**	**0.782**	**0.609**	0.375	0.011
	P-value	**< 0/0001**	0.251	**0.016**	**0.002**	**0.027**	0.207	0.970
Bazman	R	**0.740**	0.153	**0.739**	**0.719**	0.552	0.536	
	P-value	**0.006**	0.636	**0.006**	**0.008**	0.063	0.073	
Delgan	R	**0.935**	0.415	**0.776**	**0.900**	− 0.120	**0.683**	0.034
	P-value	**< 0/0001**	0.232	**0.008**	**0.000**	0.741	**0.030**	0.925
Iranshahr	R	**0.988**	**0.743**	**0.851**	**0.975**	**0.862**	− 0.130	0.160
	P-value	**< 0/0001**	**0.001**	**< 0/0001**	**< 0/0001**	**< 0/0001**	0.631	0.555

*The given numbers in bold indicate the significant correlation.

The correlations observed between EC and different ions varied significantly across the basin. In the western aquifers, moderate positive correlations were found between EC, Ca^2+^, and Mg^2+^ (0.643–0.771), suggesting their influence on overall conductivity. Bicarbonate (HCO3^−)^ showed weaker or negative correlations in most western aquifers, indicating minimal impact on EC. Conversely, the aquifers in the eastern region displayed strong positive correlations between electrical conductivity (EC) and the presence of sodium (Na^+^) and chloride (Cl^−^) ions (0.900-0.979). This indicates the presence of saline water with high levels of these ions. The aquifers in the middle region exhibited a mix of correlation patterns, suggesting a transitional zone between the western and eastern parts. The study provides insights into the distribution of cations and anions in the aquifers, which is valuable for understanding water quality and potential salinity issues.

The observed spatial variations in electrical conductivity and ion correlations indicate distinct geochemical signatures within the basin. The dominance of sodium and chloride in the eastern aquifers may be due to geological formations rich in these salts, anthropogenic inputs, or evaporation. The western region’s higher concentrations of Ca^2+^ and Mg^2+^ suggest different rock types or hydrological processes. These findings provide valuable information for characterizing groundwater quality within the basin. Although both western and eastern waters may be suitable for specific purposes, their contrasting ionic compositions dictate appropriate applications and potential limitations.

### Unraveling the relationship between groundwater level and salinity in the Jazmurian basin

#### An assessment of groundwater level fluctuations

Groundwater level fluctuations can significantly impact groundwater quality, as the water table interacts with different geological formations and alters the chemical composition of the groundwater. These changes affect hydrogeologic and water-rock interactions, contaminant transport, and saltwater intrusion, particularly in coastal areas. We created hydrographs of the aquifers to analyze their trend and behavior in groundwater levels. A hydrograph of groundwater level is a graph that displays changes in groundwater level over time, which helps in understanding the behavior of groundwater in an aquifer. Table 6 summarizes the main findings of this analysis.

**Table 6:** Statistical summary of groundwater levels for different aquifers (2001–2018).

Aquifer	Average groundwater level (meter)	Total changes in groundwater level (meter)	Annual changes in groundwater level (meter)
Jiroft	630.3	− 14.4	− 0.80
Faryab	607.7	− 14.1	− 0.79
Dashtab	2010.0	− 8.8	− 0.49
Roodbar Jiroft	407.5	− 7.6	− 0.45
Ghaleganj	383.9	− 6.6	− 0.37
Delgan	372.1	− 5.6	− 0.33
Iranshahr	497.0	− 5.4	− 0.30
Bazman	422.8	− 3.9	− 0.23
Espakeh	396.1	− 2.8	− 0.19
Chah Hashem	384.4	− 2.5	− 0.17
Esfandaghe	1689.1	− 1.4	− 0.10

Table 6 demonstrates significant variation in the average groundwater levels, ranging from 2010 m at Dashtab (the highest elevation in the basin) to 372 m at Delgan (the lowest elevation), emphasizing the pivotal role of topography in shaping groundwater dynamics.

The hydrograph survey indicates that all aquifers exhibit negative overall changes in groundwater levels, ranging from − 1.4 m in Esfandaghe to -14.4 m in Jiroft (Table 6). The analysis of annual changes in groundwater levels (Table 5) confirms a general depletion across the basin. Aquifers like those in Jiroft, Faryab, and Dashtab are at a higher risk of vulnerability owing to their more pronounced reductions.

In contrast to Noori et al.^50^, all aquifers show a consistent and worrying trend with negative values ranging from − 0.10 m in Esfandaghe to -0.80 m in Jiroft, demonstrating an annual decline in groundwater resources throughout the basin.

**Fig. 7:**
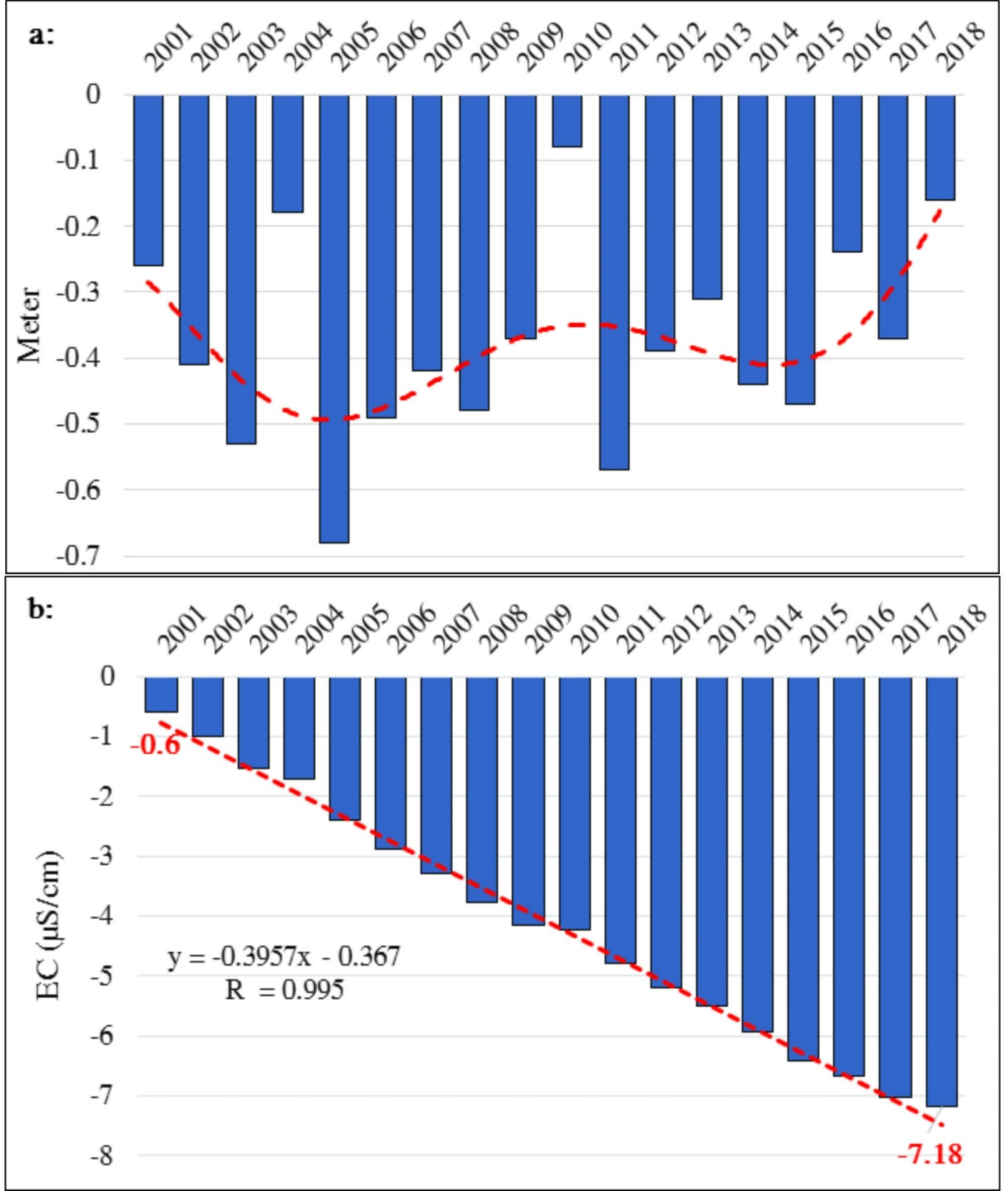
(**a**) Annual fluctuation in groundwater level (meter), and (**b**) Cumulative sum of annual changes in groundwater level (meter) in the Jazmurian Basin from 2001 to 2018.

Figure 7 displays the annual fluctuations and cumulative decline of the groundwater level in the Jazmurian Basin from 2001 to 2018. The basin experienced its most significant decline in 2005 (-0.68 m) and its most minor in 2010 (-0.08 m), with an average decline of -0.38 m per year (nearly 38 centimeters per year). This overall downward trend, resulting in a total decline of 7.18 m, indicates either over-exploitation or a natural decline in recharge that requires further investigation.

#### Assessing the relationship between electrical conductivity and groundwater level

Table 7 presents an in-depth analysis of the relationship between electrical conductivity (EC) and groundwater levels across eleven unique aquifers. It demonstrates statistical significance with a p-value of 0.05 or less, details the correlation strength and direction with an R coefficient ranging from − 1 (indicating a strong negative correlation) to 1 (indicating a strong positive correlation), and explains the proportion of variance accounted for by the regression (R^2^).

**Table 7:** Correlation between electrical conductivity (µS/cm) and groundwater level (m).

Aquifer/basin	R*	*P*-value*	Linear regression equation
Dashtab	− 0.44	0.071	y = − 56.47x + 114,581 R^2^ = 0.1893
Esfandaghe	0.56	0.045	y = 21.996x − 36,326 R^2^ = 0.3166
Faryab	0.13	0.603	y = 1.5919x + 60.23 R^2^ = 0.0173
Jiroft	− 0.81	< 0/0001	y = − 8.8241x + 6332.8 R^2^ = 0.6573
Roodbar jiroft	− **0.63**	**0.005**	y = − 52.325x + 23,212 R^2^ = 0.3988
Ghaleganj	− **0.67**	**0.002**	y = − 105.6x + 42,228 R^2^ = 0.4508
Chah Hashem	− **0.60**	**0.02**	y = − 294x + 114,354 R^2^ = 0.3649
Espakeh	− **0.82**	**< 0/0001**	y = − 258.34x + 103,599 R^2^ = 0.6798
Bazman	− **0.71**	**0.002**	y = − 246.58x + 108,388 R^2^ = 0.449
Delgan	− **0.96**	**< 0/0001**	y = − 151.82x + 77,253 R^2^ = 0.1782
Iranshahr	− 0.42	0.081	y = − 389.8x + 147,244 R^2^ = 0.9191
Jazmurian Basin	− **0.38**	**< 0/0001**	y = − 0.784x + 2477.1 R^2^ = 0.1448

*The given numbers in bold indicate the significant correlation.

Table 7 shows the relationship between electrical conductivity (EC) and groundwater level in the diverse aquifers of the basin. A basin-wide negative correlation (-0.38, *p* < 0.0001) suggests potential salinization dilution with rising groundwater levels. However, individual aquifers exhibit distinct patterns. Jiroft, Espakeh, Bazman, and Delgan stand out with strong negative correlations (R ranging from − 0.81 to -0.96, *p* < 0.0001). The linear regression equations, such as the one for Jiroft (y = -8.8241x + 6332.8, R^2^= 0.6573), indicate a notable decline in electrical conductivity (EC) as groundwater levels rise. This finding aligns with the salinization dilution hypothesis and underscores the potential influence of recharge dynamics in these aquifers. However, it’s important to note that the situation becomes more intricate when considering other aquifers.

Roodbar Jiroft, Ghaleganj, and Chah Hashem exhibit moderate negative correlations (-0.60 to -0.67) with varying significance levels (*p* = 0.002–0.005). Although the trend resembles salinization dilution, the weaker associations (lower R^2^ values) suggest potential influences from other factors besides recharge. Esfandaghe shows a unique, statistically significant positive correlation (*R* = 0.56, *p* = 0.045). The response contrasts with the basin-wide trend (equation: y = 21.996x − 36326, R^2^ = 0.3166), requiring further investigation to understand the underlying mechanisms.

Similarly, Iranshahr shows a moderate negative correlation (*R* = -0.42) that is not significant at the conventional level (*p* = 0.081). Other factors may also play a role, and further research is needed.

The equation y = -389.8x + 147,244 (R^2^ = 0.9191) suggests ambiguity in the association between groundwater depth and salinity in the studied region. In conclusion, the table shows a diverse relationship between groundwater depth and salinity in the studied region. Although a general trend of increasing electrical conductivity (EC) with decreasing groundwater levels is observed, particularly in the eastern parts of the Basin, the strength and nature of this correlation vary considerably across different aquifers. Understanding these strained relationships provides crucial insights for predicting salinization risks and informing groundwater protection and conservation strategies, such as recharge management, salinity control measures, and monitoring programs.

The presented result confirms previous findings by Ghasempour et al.^51^, Yang et al.^52^, Kaushal et al.^53^, Mirzavand et al.^54^, and Li et al.^55^, who reported a similar relationship between long-term groundwater depletion and groundwater quality.

An analysis of historical data from 2001 to 2018 revealed a complex relationship between electrical conductivity (EC) and groundwater level in the Jazmurian basin. Although there were positive correlations in specific years (e.g., 2005–2006), indicating potential mobilization of previously deposited salts and reduced dilution with rising groundwater levels, a more comprehensive analysis is required to understand the fundamental processes properly.

It is important to note that the correlation between groundwater levels and electrical conductivity (EC) is not consistently direct. There are instances, such as in 2007 and 2018, where a marked decline in groundwater levels coincided with a substantial reduction in EC. The observed decrease in Electrical Conductivity (EC) can be explained by the processes that lead to groundwater depletion, which also tend to reduce the concentration of dissolved salts in the water.

Lowering groundwater levels could concentrate existing salts in the remaining water, increasing EC. Additionally, evaporation or agricultural practices could contribute to salinization, causing an increase in EC, even with decreasing groundwater levels.

The relation between electrical conductivity (EC) and the groundwater level within the basin seems to be complex, not following a straightforward pattern, and is affected by several factors in addition to the processes of replenishment and reduction.

It is important to note that the impact of these factors on EC levels is complex and interrelated. Therefore, it is crucial to consider all relevant factors when analyzing EC levels in the basin. Salinization, which raises EC levels, can be caused by evaporation, agricultural practices, and geological formations. Conversely, increased precipitation or surface water infiltration can dilute groundwater, lowering EC levels. Sources of pollution may release salts and various chemicals into the environment, potentially influencing electrical conductivity (EC) measurements.

### Unveiling the spatially variable fingerprint of groundwater drought on salinity in the Jazmurian basin

Groundwater drought is a prolonged period of below-average groundwater levels caused by reduced recharge or increased groundwater extraction. It happens when the demand for groundwater exceeds the available supply, resulting in a depletion of groundwater reserves. This phenomenon can have vital effects on water availability and quality, affecting human and ecological systems both. This phenomenon can affect the availability and quality of groundwater, affecting human health and ecology. During groundwater droughts, water levels drop, which can lead to the intrusion of poor-quality water or the concentration of contaminants, thereby affecting water quality. Accordingly, understanding the causes and consequences of groundwater drought for proper water management is crucial. The Groundwater Risk Index (GRI) is a valuable tool for several purposes related to groundwater management. It enables identifying regions at risk of groundwater drought, assessing the impact of drought on groundwater resources, and developing water management strategies to mitigate the risk of groundwater drought.

**Fig. 8:**
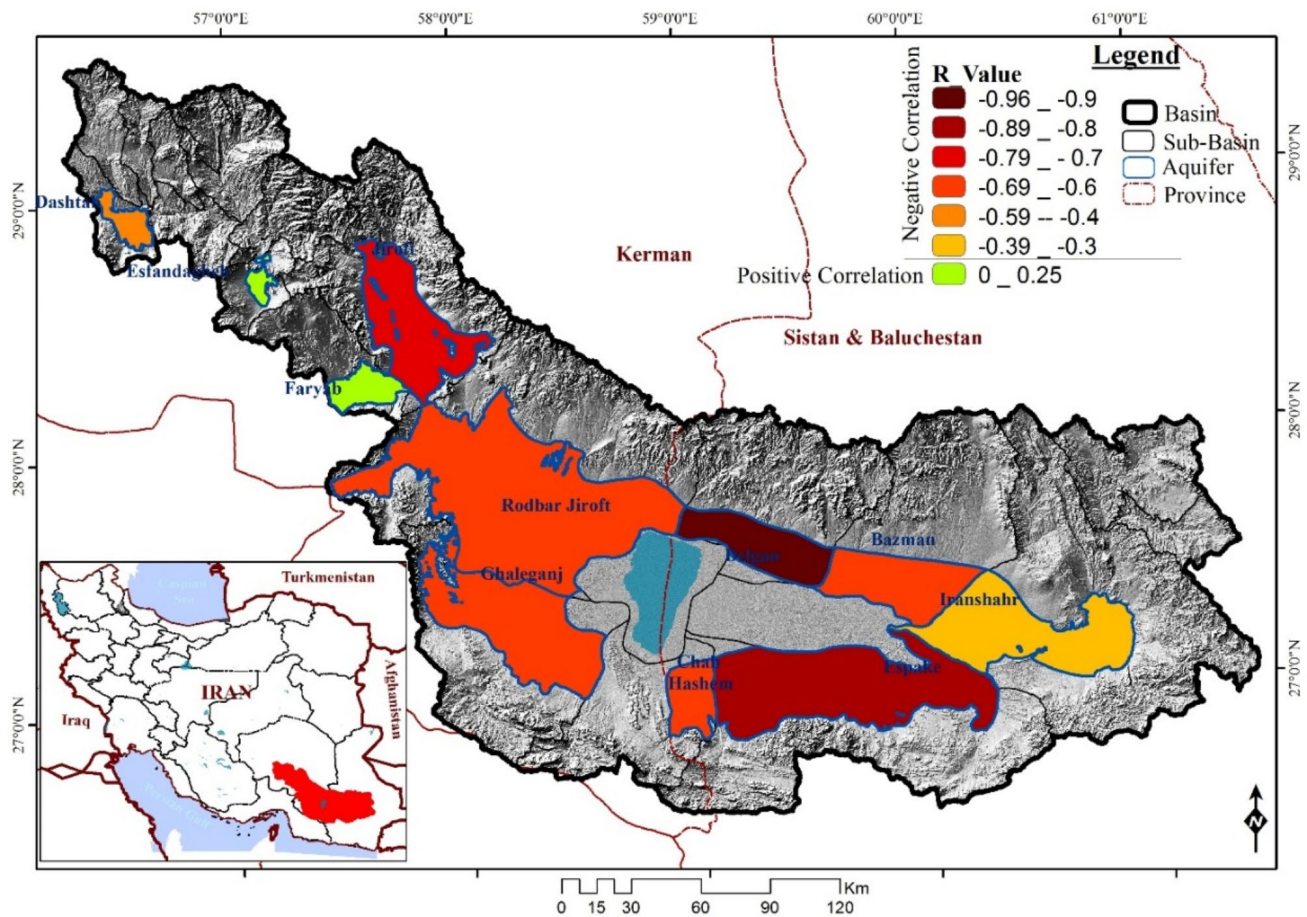
Groundwater connection - R-value relationships between EC and GRI in Jazmurian Basin’s aquifers. Maps generated using ArcGIS 10.8.1 (https://www.esri.com/en-us/arcgis/products/arcgis-desktop/overview).

Figure 8 shows the correlation values between Electrical Conductivity (EC) and the GRI index across the basin, which assesses the impact of groundwater drought on EC as a salinity indicator. Each aquifer is represented by a unique color and boundary, revealing its R-value relationship with GRI through varying color intensities. As shown in Fig. 8, the R-values range from − 0.96 to 0.25. The Delgan aquifer shows a significant negative correlation with an R-value of -0.96, indicating that a decrease in GRI (worsening drought conditions) is significantly associated with a decrease in EC. The Esfandaghe aquifer has the weakest correlation with an R-value of 0.21. The analysis suggests that changes in the GRI have less influence on EC in this area, and other factors may play a more significant role in EC variations.

Consistent with Huang et al.^56^, who evaluated groundwater drought severity and its impact on salinity in a semi-arid region of northwest China using the Groundwater Risk Index (GRI), our analysis shows a similar positive correlation between GRI values and salinity. Drought conditions can worsen salinization processes, which can compromise water quality. Li et al.^57^ used remote sensing data and the GRI to map spatiotemporal variations of groundwater drought in an arid region of northwest China, providing insights into monitoring and managing drought impacts on groundwater resources. Our findings complement previous studies by emphasizing the spatial heterogeneity in the relationship between EC and GRI across the basin. We found that coefficients shift from west to east, highlighting the intricate dynamics between hydrological variables and environmental indicators. These findings are crucial for developing effective water resource management strategies.

**Fig. 9:**
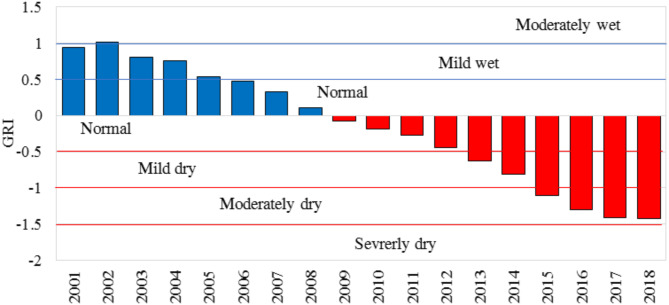
Groundwater risk index (GRI) over time in Jazmurian Basin (2001–2018).

Figure 9 displays the Groundwater Risk Index (GRI) calculated for the Jazmurian Basin during the critical eighteen-year period from 2001 to 2018.

Figure 9 shows that GRI values were generally above 0.5 from 2001 to 2004, indicating mild to moderately wet conditions. From 2005 to 2007, the GRI values decreased and fell into the mild wet and normal range. From 2009 to 2018, the GRI values became increasingly hostile, indicating a shift towards drought conditions. Notably, from 2015 to 2018, the Basin experienced moderately dry conditions with a brief dip into Severely Dry from 2017 to 2018, highlighting persistent drought challenges. The data indicates a decreasing trend in groundwater availability over the years, with more frequent and moderate drought conditions in the latter half of the period, highlighting the growing vulnerability of groundwater resources in the region. Therefore, urgent action is needed to develop effective drought mitigation and water management strategies to address this challenge.

### Understanding the drivers of groundwater quality using Gibbs diagrams, cluster analysis, and factor analysis

#### Unveiling groundwater chemistry via Gibbs diagrams

**Fig. 10:**
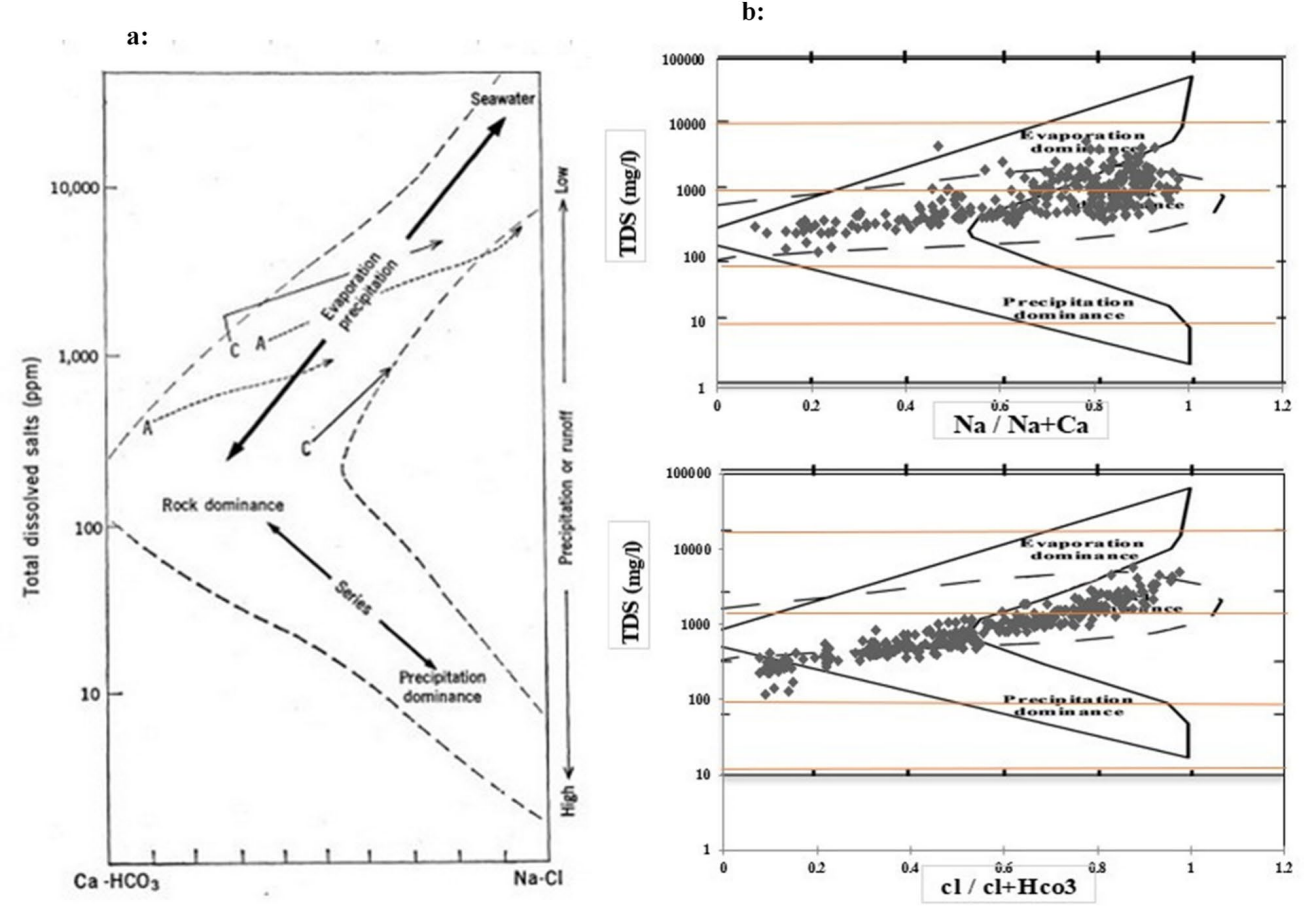
(**a**) The Gibbs diagram^40^, (**b**) The Gibbs diagram for the Jazmurian Basin.

The Gibbs diagram is a valuable tool in hydro-geochemistry for visualizing and understanding the significant factors and processes governing groundwater quality. It plots significant ion concentrations on a specific graph format (Fig. 10a) to visualize the dominant geochemical processes shaping the water’s chemical composition.

The Gibbs diagram categorizes water quality according to the dominant influence on its chemistry. The rock-water interaction zone indicates good quality water rich in minerals (Ca^2+^, Mg^2+^, Na^+^, HCO_3_^−^, SO_4_^2−^) due to mineral dissolution from the rock. The evaporation zone represents saltier water (high Na^+^/K^+^ and Cl^−^/HCO_3_^−^ ratios), which is unsuitable for drinking or irrigation due to concentrated salts. The precipitation zone, influenced by rainwater or snowmelt, represents softer water (low salinity, dominant Ca^2+^, and HCO_3_^−^) that may lack minerals essential for agriculture. Areas between these zones show a mix of influences. While natural factors such as rainfall and rock type play a role, human activities can significantly alter water chemistry through pollution, land use change, and disruption of natural water flow. Understanding this interplay is crucial to protecting our water resources.

The chemical composition of each aquifer in the Jazmurian Basin’s groundwater was investigated using Gibbs diagrams to identify the main factors that influence it (Fig. 10b). The percentage contribution of each factor was then determined and presented in Table 8 for each aquifer.

**Table 8:** Factors controlling groundwater chemistry in the Jazmurian basin.

Sub basin	Factors share (%)		
	Rock-water interaction	Evaporation	Precipitation
Dashtab	61%	39%	0%
Esfandaghe	63%	13%	25%
Faryab	85%	15%	0%
Jiroft	78%	10%	12%
Roodbar Jiroft	24%	76%	0%
Espakeh	62%	38%	0%
Ghaleganj	35%	65%	0%
Chah hashem	33%	67%	0%
Bazman	0%	100%	0%
Delgan	11%	89%	0%
Iranshahr	37%	63%	0%
Jazmurian Basin	49%	47%	4%

Following on from investigations conducted by Hosseininia and Hassanzadeh^58^ in the Rafsanjan Plain of southeastern Iran and Majidipour et al.^59^ in western Iran, our study addresses the crucial role of rock-water interaction in shaping the hydrogeochemistry of the Dashtab, Esfandaghe, Faryab, Jirft, and Espakeh aquifers (Fig. 10b). Our results are consistent with previous research and indicate that rock-water interaction exerts a significant influence, ranging from 61 to 85%, on the overall groundwater chemistry within these aquifers. This dominance is primarily due to the geological characteristics of the carbonate formations prevalent in the study area, where carbonate rocks readily dissolve in groundwater, resulting in elevated concentrations of Ca^2+^, Mg^2+^, and HCO_3_^−^ ions. By validating and extending previous observations, our study improves the understanding of the hydrogeochemical processes governing groundwater quality in carbonate-dominated aquifer systems, providing valuable insights for water resource management and environmental protection efforts in similar geological contexts.

Our analysis highlights the importance of evaporation as the main factor influencing groundwater salinity in the Bazman, Delgan, Roodbar Jiroft, Chah hashem, Ghaleganj, and Iranshahr aquifers, in agreement with the findings of Ahmadi et al.^60^ and Mohammadi Arasteh and Shoaei^61^, who studied aquifers in arid regions of Iran. Our study confirms that evaporation significantly contributes to groundwater salinity in these aquifers, accounting for between 63% and 100% of the total influence. This dominance is due to the arid or semi-arid nature of the regions hosting these aquifers, characterized by high evaporation rates concentrating salts in the remaining water, resulting in elevated Na^+^ and Cl^−^ concentrations. The research presented here confirms and extends previous findings on hydrogeochemical processes that drive salinity in groundwater resources, particularly in arid environments. The insights gained from this research can be valuable for developing sustainable water management strategies in similar geographical settings. In line with the findings of Eslami et al.^62^ regarding the positive influence of increased precipitation on groundwater quality in the Espakeh and Jiroft aquifers, our study strengthens this relationship. Consistent with previous research, our analysis shows that precipitation plays a secondary role in groundwater chemistry in these aquifers, contributing 25% and 12% of the total influence, respectively. These aquifers, which experience higher rainfall than the others, facilitate the leaching of ions from soils and rocks into groundwater, resulting in lower salinity levels and the prevalence of Ca^2+^ and HCO_3_^−^ ions. In addition, the observations of Narany et al.^63^ in the Amol-Babol Plain further support our findings for the Jiroft aquifer, highlighting the impact of higher precipitation on groundwater characteristics in the region. By corroborating and extending previous research, our study improves the understanding of the hydrogeochemical processes governing groundwater quality in areas influenced by different precipitation patterns, providing valuable insights for water resource management in similar geographical contexts.

**Fig. 11:**
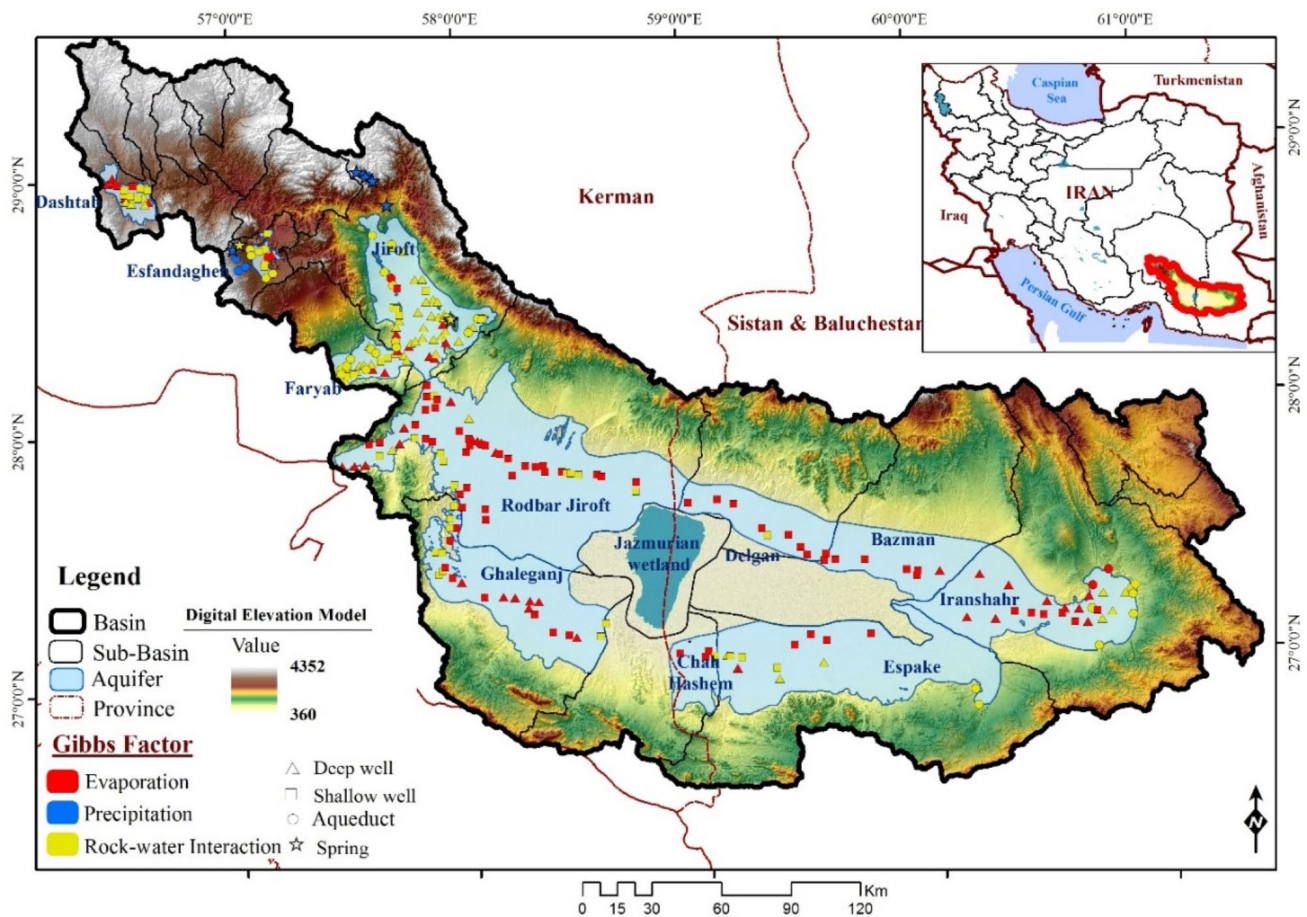
Factors shaping groundwater chemistry in different water sources of the Jazmurian Basin. Maps generated using ArcGIS 10.8.1 (https://www.esri.com/en-us/arcgis/products/arcgis-desktop/overview).

The groundwater chemistry analysis by resource type (Fig. 11) reveals that in Dashtab, rock-water interaction dominates in deeper aquifers (DW), while shallow wells (SW) display some influence from evaporation. Springs (S) exhibit a mix of rock water and precipitation. Deep wells in Esfandaghe and Faryab primarily exhibit rock-water interaction, while shallower resources (G and SW) experience some evaporation. DW and G resources showcase rock-water interaction with minor evaporation, while SW wells see higher evaporation in Jiroft, suggesting surface process influence. In Roodbar Jiroft and Espakeh, SW wells have experienced combined rock water and evaporation influence, possibly due to regional factors. It is important to note that all evaluations are presented objectively and without bias. Deeper resources (DW) in Espakeh primarily exhibit rock-water interaction. In Ghaleganj, both shallow and deep resources are mainly influenced by rock-water interaction, with a possible minor impact from evaporation in shallow wells. Chah Hashem displays mixed influences of rock water and evaporation in shallow and deeper resources. In Ghaleganj, both shallow and deep resources are mainly influenced by rock-water interaction, with a possible minor impact from evaporation in shallow wells. Chah Hashem displays mixed influences of rock- water and evaporation in shallow and deeper resources. Analogous Jiroft, deeper resources in Iranshahr exhibit rock-water influence with minor evaporation, while shallow wells are more significantly impacted by evaporation, indicating surface processes. Therefore, deep wells (DWs) often demonstrate dominance of rock-water interactions due to access to deeper aquifer zones less affected by surface processes such as evaporation. Gu et al.^64^ study in North China confirms that geogenic processes, specifically rock weathering and ion exchange, are the only mechanisms that control groundwater chemistry in deep aquifers.

The influence of Ghanat (G) varies depending on depth, location, and connection to specific aquifer sections. Springs (S) are often affected by precipitation or shallow aquifer zones, which can result in some rock-water interaction. Shallow wells (SW) are more susceptible to evaporation due to their proximity to the surface.

Our study builds upon the insights provided by Teramoto et al.^65^ and Zeng et al.^66^ to elucidate further the hydrogeochemical processes that shape groundwater quality within the Jazmurian Basin. Consistent with previous research, our findings highlight the prevalence of rock-water interaction as the primary process observed in 49% of samples across the basin. However, the influence of rock-water interaction varies significantly among aquifers and resource types. Evaporation is a significant factor, especially in aquifers with shallow wells (SW) and those in the central and eastern regions, accounting for 47% of samples. In contrast, precipitation has a minimal impact overall, with only 4% of samples affected by its contribution, mainly in springs (S) and deep wells (DW) within the Esfandaghe and Jiroft Aquifers. These findings support the work of Teramoto et al.^65^, who emphasized the importance of understanding groundwater quality through understanding the mechanisms underlying rock-water interactions. Similarly, Zeng et al.^66^ highlighted that the chemical composition of groundwater is constantly influenced by water-rock interaction, evaporation, and concentration, resulting in a dynamic equilibrium. Our research offers a comprehensive understanding of the hydrogeochemical dynamics driving groundwater quality in the Jazmurian Basin through the integration of multiple studies that provide valuable insights for effective water resource management strategies in arid environments.

#### Cluster analysis to identify common drivers of groundwater chemistry

**Fig. 12:**
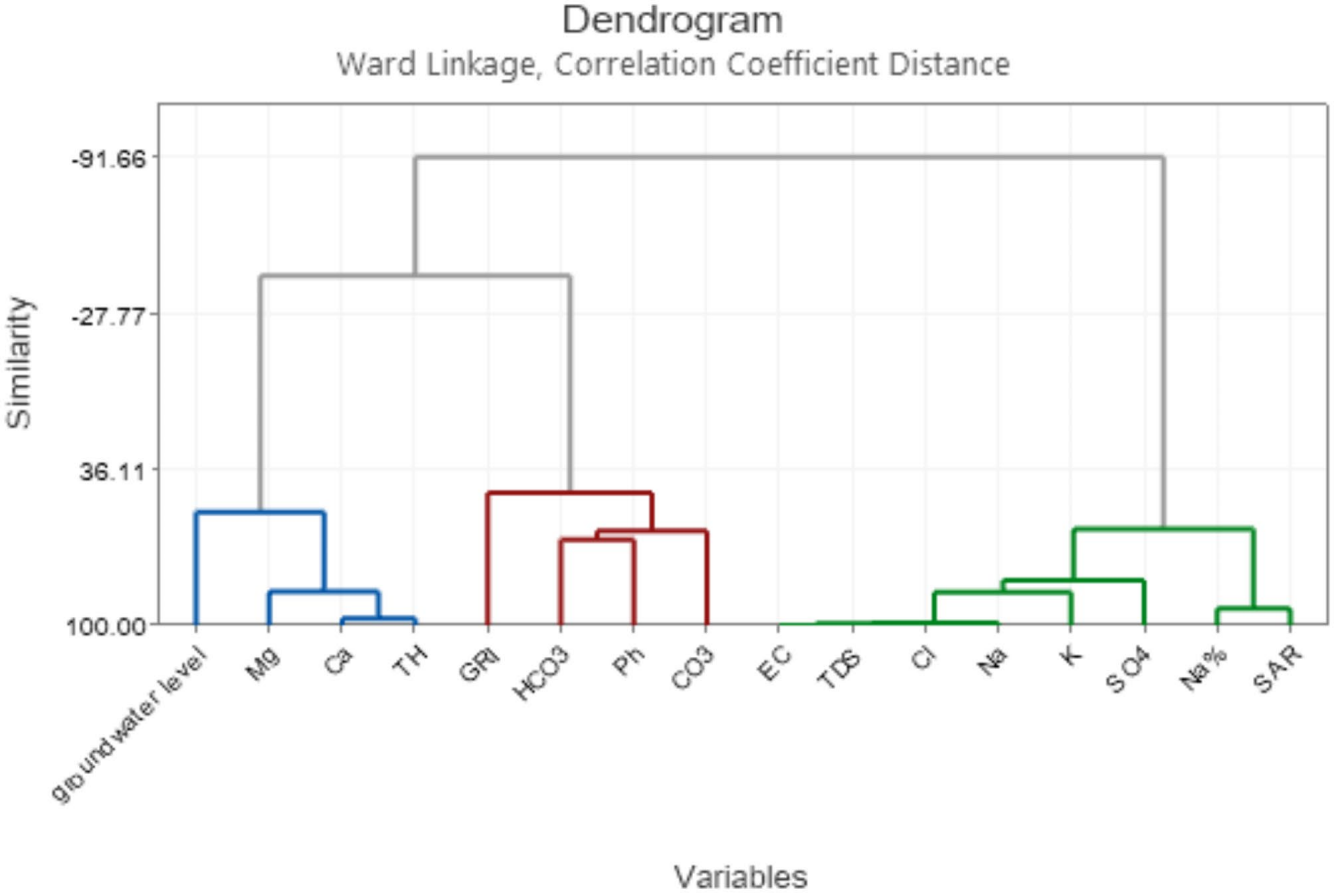
Cluster analysis of physicochemical parameters of groundwater.

Cluster analysis, a statistical technique commonly employed in environmental studies, was performed to categorize groundwater samples based on physicochemical parameters. This approach aids in identifying similarities and differences among various water quality indicators, facilitating the formation of distinct clusters. The resulting clusters provide valuable insights into the factors contributing to overall groundwater quality and potential geological influences. Figure 12 represents the result of cluster analysis, which grouped the data into three clusters based on variable similarity.

Cluster 1, consisting of groundwater level, Mg^2+^, Ca^2+^, and TH variables, probably represents groundwater influenced by the underlying rock formations of the basin. Deeper groundwater levels could indicate interaction with deeper aquifers within limestone or dolomite formations, possibly contributing to higher hardness (TH) due to increased calcium and magnesium (Mg^2+^, Ca^2+^). Understanding the local geology, particularly the distribution of different rock types, will be crucial in predicting the characteristics and distribution of the water in this cluster.

Cluster 2, which includes variables such as GRI, HCO_3_^-^, CO_3_^2-^, and pH, indicates groundwater with good recharge potential and favorable quality.

The presence of bicarbonate and carbonate ions (HCO₃⁻, CO₃^2^⁻) suggests an active recharge—likely due to infiltration from rivers and streams in the surrounding Zagros and Makran mountain ranges. The low drought susceptibility (GRI) and near-neutral pH further affirm the suitability of this water for various purposes. To ensure the long-term sustainability of this valuable resource, safeguarding recharge processes within this cluster—potentially linked to mountain runoff—is crucial.

Cluster 3 is a group of groundwater samples that have high salinity levels. It is made up of EC, Na^2+^, K^+^, SO_4_^2-^, Cl^-^, TDS, Na%, and SAR. The elevated values of electrical conductivity (EC) and the concentrations of sodium (Na^+^) and chloride (Cl^-^) suggest that the water may be brackish or saline, which can make it unsuitable for direct consumption without treatment.

High levels of sodium (Na%) and sodium adsorption ratio (SAR) may cause damage to soil structure, leading to potential irrigation challenges. It is crucial to understand the sources of salinity in this cluster, whether controlled by natural origins, such as the presence of evaporites like gypsum, or influenced by agricultural practices. Sustainable water management in this cluster will require careful treatment and responsible use to minimize environmental impact.

Comparison of our results with similar studies in arid and semi-arid regions of Iran, such as those by Rahimi Shahid et al.^67^, Esmaeili-Vardanjani et al.^68^, and Barzegar et al.^69^, as well as the studies by Garba et al.^43^ and Hardle and Simar^70^, highlights the usefulness of cluster analysis in identifying spatial variations in groundwater quality and understanding the influences of natural processes and human activities. The study by Liu et al.^71^ emphasizes the importance of evaluating both geological and human factors when assessing groundwater quality and its suitability for different uses in comparable environmental contexts.

Consequently, the Results underline the usefulness of cluster analysis as a tool for effective water quality management in arid and semi-arid regions, helping to develop targeted strategies for the sustainable use of groundwater resources.

#### Quantify underlying factors with factor analysis

Groundwater, a vital resource, is often vulnerable to contamination from various sources. Understanding the factors influencing its quality is crucial for sustainable management and protection. Factor analysis, a powerful statistical technique, can be applied in this endeavor. In the context of groundwater quality, it analyses various water chemistry parameters, looking for hidden patterns. These patterns, known as factors, represent common influences on different parameters and highlight the underlying processes affecting water quality. Table 9 highlights the most critical factors influencing groundwater quality in the Jazmurian Basin.

**Table 9:** Factor loadings and variability for groundwater parameters in the Jazmurian basin.

Parameter	F1	F2	F3	F4
groundwater level (m)	− 0.44	0.352	0.01	**0.592**
GRI	0.187	− **0.23**	0.003	0.173
EC (µS/cm)	**0.963**	0.265	− 0.039	− 0.014
Na^+^	**0.981**	0.166	− 0.026	− 0.095
Mg^2+^	0.131	**0.823**	0.054	0.339
Ca2+	0.16	**0.908**	− 0.157	− 0.116
K^+^	**0.869**	− 0.171	− 0.106	0.149
SO_4_^2−^	**0.73**	0.531	− 0.073	− 0.116
Cl^−^	**0.948**	0.247	− 0.134	− 0.01
HCO_3_^−^	− 0.163	− 0.132	**0.464**	− 0.054
CO_3_^2−^	0.092	− **0.414**	0.377	0.048
pH	− 0.182	− 0.2	**0.514**	0.008
TDS	**0.961**	0.266	− 0.035	− 0.015
Na%	**0.765**	− 0.401	0.237	− 0.348
SAR	**0.839**	− 0.292	0.168	− 0.143
TH	0.159	**0.983**	− 0.046	0.083
Eigenvalue	6.936	3.725	0.629	0.526
Variability (%)	41.588	22.863	4.836	4.42
Cumulative %	41.588	64.451	69.287	73.708

Factor 1 (41.588% variability, Eigenvalue 6.936) is associated with high salinity and ionic strength in groundwater. The high eigenvalue indicates its prominent role in explaining the dataset variance (41.588%). The high loadings of significant ions, including Na^+^, Cl^−^, EC, and TDS, suggest that this phenomenon arises from the concentration of salts in the water. The primary contributors to the heightened salinity and ionic strength are a combination of factors: an arid climate, salt, and clay crusts, limited water resources, and potentially geological formations such as gypsum evaporites within the basin. The arid climate leads to water loss, intensifying the concentration of existing salts and elevating salinity levels. Salt and clay crusts act as reservoirs, releasing salts intermittently and further impacting salinity. Furthermore, poorly water-bearing rock formations impede water flow, exacerbating salt accumulation.

Factor 2 focuses on the mineral content and hardness of groundwater. The moderate eigenvalue (6.94) reflects its importance in explaining the variance of the dataset. High loadings for magnesium (0.823), calcium (0.908), and total hardness (0.983) in the water signal the influence of particular rock formations. Higher levels of Mg^2+^ and Ca^2+^ contribute to hardness, and their presence could indicate interaction with limestone, dolomite, or other carbonate rocks known to be present in the basin.

In addition, specific fertilizer or irrigation practices may also increase Mg^2+^ and Ca^2+^ levels and contribute to hardness. Overall, the weathering of the host rock and agricultural practices are the activities that shape Factor 2.

Factor 3 is a significant contributor, responsible for 4.84% of the variation in groundwater quality in the Jazmurian Basin. This factor is identified by high levels of bicarbonate (0.464) and carbonate (0.377), along with an almost neutral pH (7.2).

These observations indicate active recharge processes, likely resulting from surface water infiltration or the dissolution of carbonate formations. The hydrogeochemical characteristics are favorable, rendering the water suitable for various purposes. Notably, the near-neutral pH effectively enhances the dynamic replenishment mechanism, emphasizing the abundance of bicarbonate and carbonate ions.

Factor 3 thus highlights the critical role of recharge processes in maintaining the basin’s healthy groundwater reserves.

Understanding the intricate interplay between mountain runoff, river systems, and infiltration patterns is paramount to preserving these vital recharge areas. By carefully protecting these areas, we can ensure the continued availability of high-quality groundwater for present and future generations.

Factor 4, which explains 4.42% of the variance (eigenvalue 0.526), reveals a complex interplay between specific ion concentrations and groundwater levels. While a moderate loading on the groundwater level (0.592) suggests a relationship, the scattered and weaker loadings on individual ions suggest a multifaceted relationship. The lower eigenvalue of these factors highlights their relatively low impact on the water. Several potential driving forces may be at play: Seasonal variations: Rainfall patterns could influence specific ions such as nitrates or sulfates through seasonal agricultural run-off or weathering processes.

Geological heterogeneity within the Jazmurian Basin could result in different ion concentrations depending on the underlying rock formations encountered by the groundwater. Fluctuations in groundwater levels could mobilize previously deposited contaminants, affecting specific ion concentrations or altering flow patterns, leading to interactions with different rock layers and their associated minerals.

Comparing our findings with similar studies conducted in Iran, such as those by Eslami et al.^62^, Mohammadi Arasteh and Shoaei^61^, and Khalili and Asadi^72^, highlights the effectiveness of factor analysis in identifying the primary factors influencing groundwater quality, including natural processes and human activities. In addition, studies by Bahrami and Zarei^73^, Jabbari et al.^74^, and Karimi et al.^75^ highlight the importance of considering spatial variation and potential health risks associated with groundwater contamination, further emphasizing the relevance of factor analysis in water quality management and risk assessment.

To summarize, the factor analysis results are consistent with previous studies conducted in Iran and identify vital factors shaping groundwater quality in the Jazmurian Basin. Similar to the findings of Eslami et al.^62^ and Mohammadi Arasteh and Shoaei^61^, our factor 1 highlights the influence of geological factors and anthropogenic activities on salinity. However, our study also emphasizes the impact of arid climatic conditions on salinity levels.

In addition, Factor 2 reflects the observations of Eslami et al.^62^ on the influence of rock weathering and agricultural practices on groundwater mineral content and hardness. Conversely, our Factor 3 results, which indicate active recharge processes and favorable hydrogeochemical properties, differ from studies such as Khalili and Asadi^72^, which focused on identifying pollution sources. Furthermore, our result suggests a complex relationship between specific ion concentrations and groundwater levels in Factor 4, which contrasts with the emphasis on health risk assessment in studies by Jabbari et al.^74^ and Karimi et al.^75^.

Despite similarities in identifying key drivers of groundwater quality, our study provides unique insights into the complex interplay of natural and anthropogenic factors specific to the Jazmurian Basin.

## Conclusion

This study employed a multifaceted approach, encompassing diverse techniques like physicochemical analyses, trend detection, geochemical fingerprinting, and drought impact assessment, to unravel the intricate tapestry of groundwater quality within the arid Jazmurian Basin. Key findings reveal significant spatial and temporal variations in salinity, hardness, and sodium hazard across aquifers, highlighting the need for tailored management strategies.

The ongoing rise in electrical conductivity (EC), specifically in the eastern area, highlights the importance of constant monitoring and targeted actions. The nuanced relationship between groundwater level and salinity demands site-specific management beyond generalized approaches while understanding the spatially variable fingerprint of drought empowers targeted interventions for salinity mitigation.

Geochemical investigations unveil the interplay of rock-water interaction, evaporation, and precipitation, painting a vivid picture of aquifer-specific influences. These insights, including the identifying salinity drivers and the complex relationship between ions and groundwater level, pave the way for sustainable water management. Cluster analysis further refines this understanding by identifying three distinct water quality profiles, each demanding differentiated management approaches.

Factor analysis highlighted the considering multiple factors beyond the chemical composition when evaluating groundwater quality and vulnerability. This statistical method unveils the underlying factors influencing water quality, emphasizing the importance of addressing salinity risks, mitigating anthropogenic influences, and protecting recharge zones for long-term sustainability.

As a whole, this research offers valuable insights for sustainable water management in arid environments. By identifying potential salinization risks, guiding water quality monitoring efforts, and informing strategies for responsible water use, it contributes to a brighter future for the precious groundwater resources of the Jazmurian Basin and arid regions globally. Future investigations could delve deeper into specific anthropogenic influences and further refine our understanding of the unique geochemical processes shaping each aquifer system, ultimately enhancing groundwater resource management worldwide.

## Data Availability

The authors confirm that all data generated or analyzed during this study are included in this published article.
